# Mechanistic insights into the selective targeting of P2X3 receptor by camlipixant antagonist

**DOI:** 10.1016/j.jbc.2024.108109

**Published:** 2024-12-18

**Authors:** Trung Thach, KanagaVijayan Dhanabalan, Prajwal Prabhakarrao Nandekar, Seth Stauffer, Iring Heisler, Sarah Alvarado, Jonathan Snyder, Ramaswamy Subramanian

**Affiliations:** 1 Department of Biological Sciences, Purdue University, West Lafayette, Indiana, USA; 2 Elanco Animal Health, Greenfield, Indiana, USA; 3 Weldon School of Biomedical Engineering, Purdue University, West Lafayette, Indiana, USA

**Keywords:** cryo-EM, P2X3, ion channel, camlipixant, receptor

## Abstract

ATP-activated P2X3 receptors play a pivotal role in chronic cough, affecting more than 10% of the population. Despite the challenges posed by the highly conserved structure of P2X receptors, efforts to develop selective drugs targeting P2X3 have led to the development of camlipixant, a potent, selective P2X3 antagonist. However, the mechanisms of receptor desensitization, ion permeation, and structural basis of camlipixant binding to P2X3 remain unclear. Here, we report a cryo-EM structure of camlipixant-bound P2X3, revealing a previously undiscovered selective drug-binding site in the receptor. Our findings also demonstrate that conformational changes in the upper body domain, including the turret and camlipixant-binding pocket, play a critical role: turret opening facilitates P2X3 channel closure to a radius of 0.7 Å, hindering cation transfer, whereas turret closure leads to channel opening. Structural and functional studies combined with molecular dynamics simulations provide a comprehensive understanding of camlipixant's selective inhibition of P2X3, offering a foundation for future drug development targeting this receptor.

Neuronal signaling that prompts coughing is well documented and is primarily mediated by ligand-gated ion channels known as P2X3 receptors (1, 2). These receptors, comprising an extracellular ligand-binding domain and a membrane-spanning ion channel domain, swiftly convert extracellular chemical signals into transmembrane ionic currents, facilitating rapid intercellular signaling and chemosensation (3, 4). Purinergic P2X3 receptors gated by ATP exist in both homotrimeric (comprising three P2X3 subunits) and heterotrimeric (comprising one P2X2 subunit and two P2X3 subunits, termed P2X2/3) configurations. Although P2X3 has been implicated in pathological cough, P2X2/3 also plays a role in taste transmission (1, 5). Antagonists targeting P2X3 hold promise for chronic cough treatment by attenuating neuronal hypersensitivity. However, designing drugs that selectively target P2X3 over P2X2/3 or other P2X receptors is challenging owing to the highly conserved structures of these receptors. For example, gefapixant, a negative allosteric modulator of P2X3 receptors, has shown encouraging results in clinical trials targeting refractory chronic cough. Nonetheless, gefapixants can induce side effects, notably taste alteration or loss (6). This phenomenon arises from the dual action of gefapixant, which affects P2X3 associated with refractory chronic cough and P2X2/3 involved in taste signal transmission (6, 7).

Recently, camlipixant has emerged as a highly selective P2X3 antagonist with significantly higher potency against P2X3 than against P2X2/3 (8). Camlipixant shows potential for cough relief with a minimal impact on taste perception and is currently undergoing phase III clinical trials (NCT05599191). Despite these promising results, the structural basis underlying the selective targeting of P2X3 by camlipixant remains unclear. Although the crystal structures of P2X3 have elucidated common mechanisms, such as ATP and antagonist binding and gating within the P2X receptor family (9, 10, 11, 12), questions remain regarding the mechanism that makes camlipixant specific to P2X3. For instance, what are the structural determinants of camlipixant's preference for P2X3 over P2X2/3?

Considering the importance of P2X receptors as drug targets, we identified and mapped the binding site of camlipixant on P2X3 using single-particle cryo-EM. Our results shed light on the structural basis of the inhibitory action of camlipixant on P2X3. Our findings, supported by biochemical and biophysical experiments and molecular dynamics (MD) simulations, revealed a cryptic drug-binding site for camlipixant that disrupts the conformational changes associated with P2X3 receptor inhibition. Structural insights into the camlipixant-bound P2X3 complex provide a foundation for future drug discovery efforts that target the P2X receptor family.

## Results

### Cryo-EM structure of camlipixant-bound P2X3 reconstituted into peptidisc

To explore camlipixant binding at the structural level, our initial strategy involved establishing an Expi293F GnTI-stable cell line expressing GFP-P2X3 using the PiggyBac transposon system (Fig. S1, *A* and *B*). Viability and GFP fluorescence monitoring indicated a higher P2X3 expression at 30 °C than at 37 °C (Fig. S1, *C* and *D*). Calcium influx assays confirmed the functional expression of P2X3 in the cells (Fig. S2*A*). Camlipixant-treated cells exhibited a dose-dependent decrease in function upon addition of ATP–Mg^2+^ (Fig. S2*B*). Camlipixant inhibited ATP-activated P2X3-mediated Ca^2+^ ion transfer in a concentration-dependent manner, with an IC_50_ of 55.05 nM (Fig. S2*C*).

Next, we determined the cryo-EM structures of the P2X3 receptor complexed with camlipixant. However, challenges arise owing to the preferred orientation issues in the purified state with *n*-dodecyl-β-d-maltoside (DDM) detergent. Resolution of the transmembrane domains was elusive (Fig. S3, *A–D*). To resolve the full-length receptor complexed with camlipixant, we employed the peptidisc approach to stabilize the receptor and bind it to camlipixant (13, 14, 15). Notably, reconstitution into the peptidisc significantly improved receptor distribution on the cryo-EM grid (Fig. S3, *E* and *F*). The cryo-EM density exhibited particularly well-defined features, with a local resolution ranging from 3.0 to 3.2 Å resolution (Fig. S4 and Table S1). We observed an additional density surrounding the P2X3 transmembrane helices, suggesting the presence of peptidiscs (Fig. S5, *A* and *B*). We unambiguously rebuilt and assigned side chains to all residues in camlipixant-binding pocket (Fig. S5, *C–E*). Superimposing our structure onto X-ray crystallography–determined structure of P2X3 (Protein Data Bank [PDB] ID: 5SVJ) suggested that no major structural rearrangements were observed in transmembrane helices (RMSD of 0.50 Å). This observation strongly suggests that peptidisc stabilization preserves the structural integrity of P2X3.

**Figure 1:**
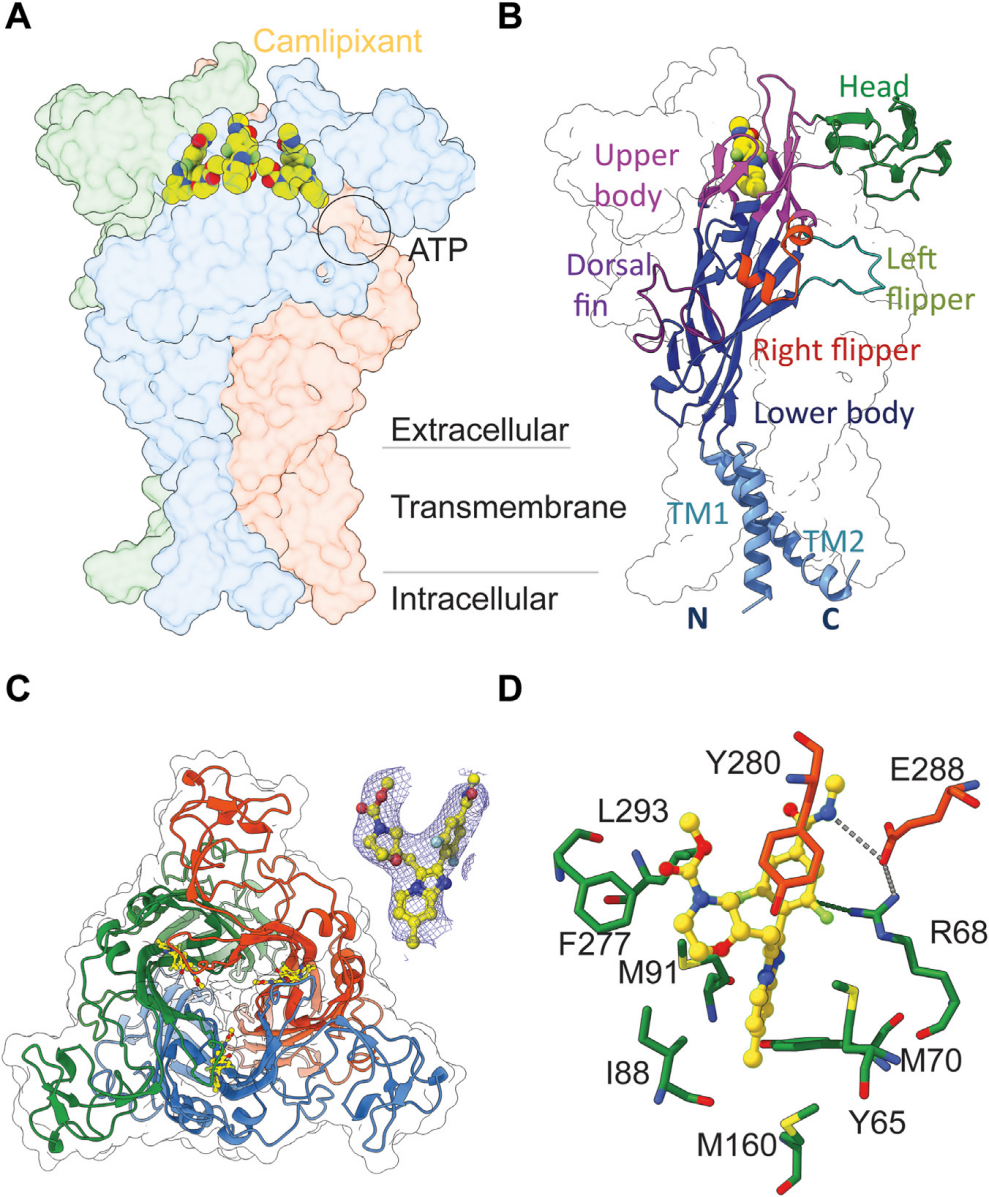
**Cryo-EM structures of the camlipixant (Cam)-bound P2X3 receptor, reconstituted into peptidisc.***A* and *B*, the overall structure of the P2X3–Cam complex reconstituted into peptidisc, with each subunit color-differently, is depicted. The protomers of the trimer are colored *blue*, *orange*, and *forest*. Surface transparency was adjusted to 90%. The P2X3 monomer adopts a dolphin-like shape, with each region of the P2X3 protein depicted in *colored cartoon* representation according to this shape. Cam (shown as *spheres*) binds to the upper body domain of P2X3. *C*, representations of the structure viewed perpendicular to the membrane from the extracellular side. The map for Cam is shown and contoured at 3.0 σ. *D*, an insight view of the interactions between Cam and P2X3. The C-backbone of Cam is shown in *yellow color*. Hydrogen bond is shown as a *dashed line*.

The P2X3 structure consists of a homotrimer (Fig. 1*A*). A single subunit of the P2X3 receptor displayed a distinctive “dolphin-like” shape, akin to the broader P2X receptor family (Fig. 1*B*). Camlipixant binds to the pocket above the ATP-binding site formed by the upper body domain between the adjacent subunits (Fig. 1, *A* and *B*). This drug-binding pocket is encircled by residues that predominantly protrude from the β-strands (β3, β4, β5, β13, and β14) (Figs. 1, *B* and *C* and S6, and Table S2). Residues M70, M91, F277, and L293 (subunit A) form a complex network of contacts favoring nonpolar environments, indicating a predominantly hydrophobic binding interface between the receptor and camlipixant. Specifically, R68 (subunit A) and E288 (subunit C) recognize camlipixant by cation–π and charge–charge interactions with the difluorophenyl ring and N04, respectively, acting as a lid in the binding process (Figs. 1*D* and S5, *D* and *E*). The evolutionary conservation of positions of residues in the P2X3 structure was estimated based on the phylogenetic connections among homologous sequences using Consurf (16), suggesting that these camlipixant-bound residues are moderately conserved in P2X3–7 but distinct from those found in P2X2 receptors (Figs. S6–S8 and Table S2). This reinforces the highly selective targeting of P2X3 over P2X2/3 receptors by camlipixant (8).

### The camlipixant-binding site appears highly selective in P2X3 receptor

**Figure 2:**
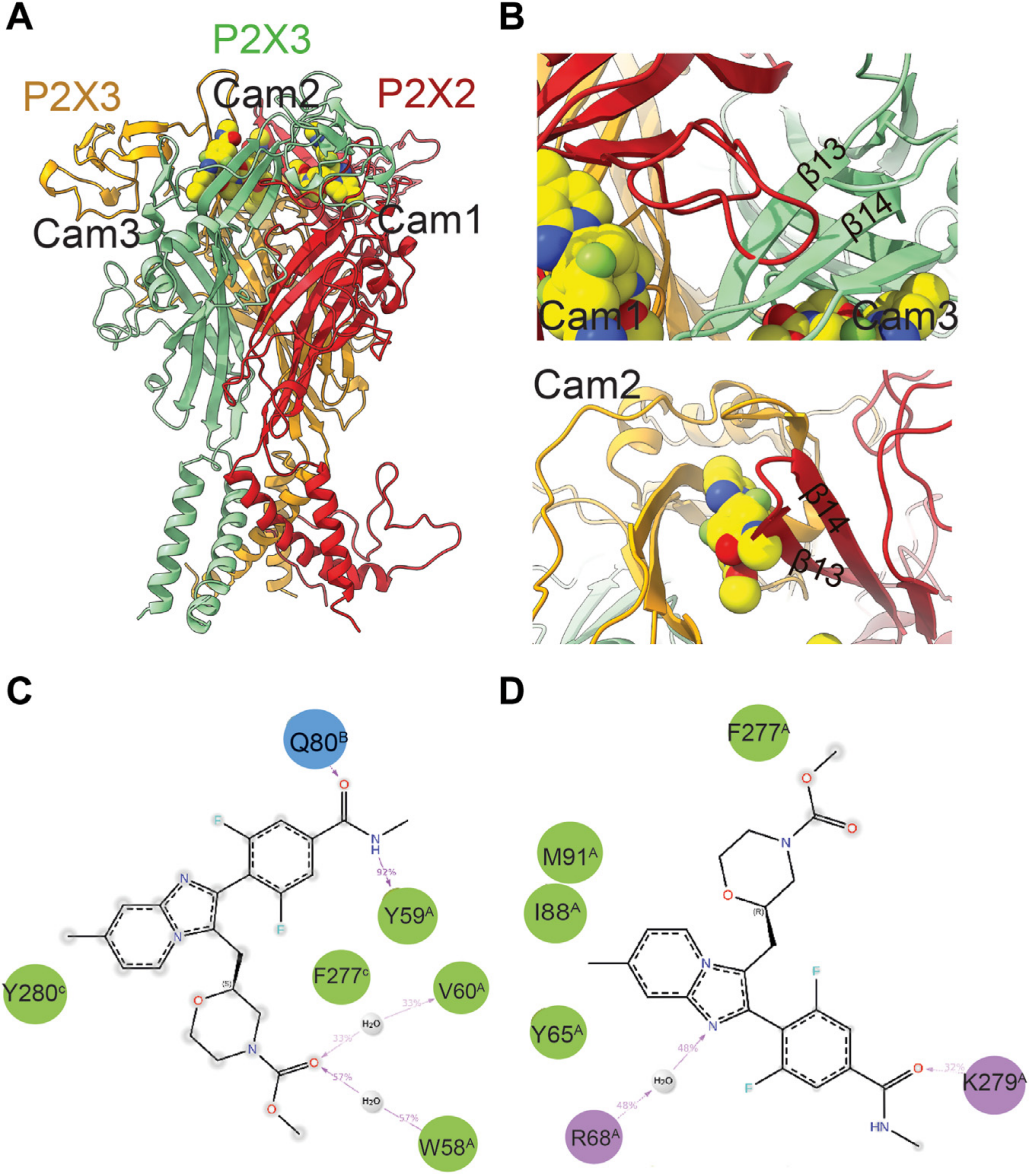
**Camlipixant (Cam) exhibits selective targeting of the P2X3 over the P2X2/3 receptor.** The illustration depicts a model of the P2X2/3–Cam complex, wherein the P2X2/3 receptor is overlaid onto the P2X3 receptor shown in a *cartoon diagram*. The Cam ligand is represented as *spheres*. *B*, Cam1 and Cam2 are situated at the interface of P2X2, whereas Cam3 binds within the interface of P2X3. *C* and *D*, a schematic diagram outlines the interactions between either Cam1 or Cam2 with P2X2/3 post molecular dynamics simulations of the complex.

Utilizing our resolved P2X3–camlipixant structure in this study, we modeled both the P2X2 structure and P2X2/3 heterotrimer binding to camlipixant (Fig. 2*A*). Given the plethora of human P2X2 receptor isoforms, we chose isoform K because of its high sequence identity to P2X3 (Fig. S8). Notably, we observed discrepancies not only in the sequence but also in the structure of the camlipixant-binding pocket. The loop between β4 and β5 in P2X2 is longer and clashes with β13 and β14 in the adjacent subunit when bound to camlipixant (Cam1; Fig. 2, *A* and *B*). In another binding site, the β13 and β14 regions shifted forward toward the adjacent subunit, thereby narrowing the pocket by approximately 5%, potentially inducing significant alterations in the camlipixant interaction (Cam2; Fig. 2*C*). Furthermore, we used the camlipixant-bound P2X2/3 structure as the initial conformation for the MD simulations. Trajectories were analyzed to assess ligand stability and protein conformational changes. The lowest binding free energy was obtained after a 100 ns conformational sampling process that explored all potential binding modes of the receptor and ligand conformations (Fig. S9 and Table S3). We noted a considerable increase of approximately 36% in the MMGBSA-binding free energy at both binding interfaces between the P2X2–P2X3 (Cam1) and (Cam2) subunits compared with the camlipixant-bound P2X3 (Table S3). The camlipixant is mostly bound to the outside of the upper vestibule; the hydrophobic group is exposed to a solvent environment during the simulation, indicating that camlipixant could not bind well to the P2X2/3 receptor interface (Fig. 2, *C* and *D*).

To explore the potential crosstargeting of camlipixant and other antagonists among P2X receptors, we conducted flexible docking and identified interactions during MD simulation in a matrix. Our data indicate that camlipixant exhibits binding affinity to P2X3 over P2X4 and P2X7, as evidenced by the lower free energy values and docking scores (Fig. S10, *A* and *B* and Table S3). The hydrophobic group fits into the lumen inside the P2X3-binding pocket, with the carboxyl group interacting mainly with the charged residues. In contrast, the interactions between camlipixant and P2X4 showed fewer hydrogen bonds and hydrophobic interactions. The carboxyl group of camlipixant was exposed to the hydrophilic solvent outside the pocket, potentially exposing it to the solvent in interactions with P2X7 (Fig. S10, *C–E*). It is worth noting that the intersubunit cavity formed by β13 and β14 in the upper body domain is wider in P2X3 than in zebrafish P2X4 and panda P2X7. The camlipixant-binding pocket volumes measured were 1203 Å^3^ (P2X3), 1034 Å^3^ (P2X4), and 1183 Å^3^ (P2X7). Although the structures of P2X1/5/6 receptors have not been reported, the volume of the binding pocket differs from that of P2X3 because of variations in the key residues (Fig. S7 and Table S2). These findings suggest that narrowing of the intersubunit space is specific to the P2X3 receptor. Thus, differences in the size of the hydrophobic pocket and antagonist drugs are critical for selective binding to P2X3 receptors.

### The ion entry and gate are closed during camlipixant-bound state

**Figure 3:**
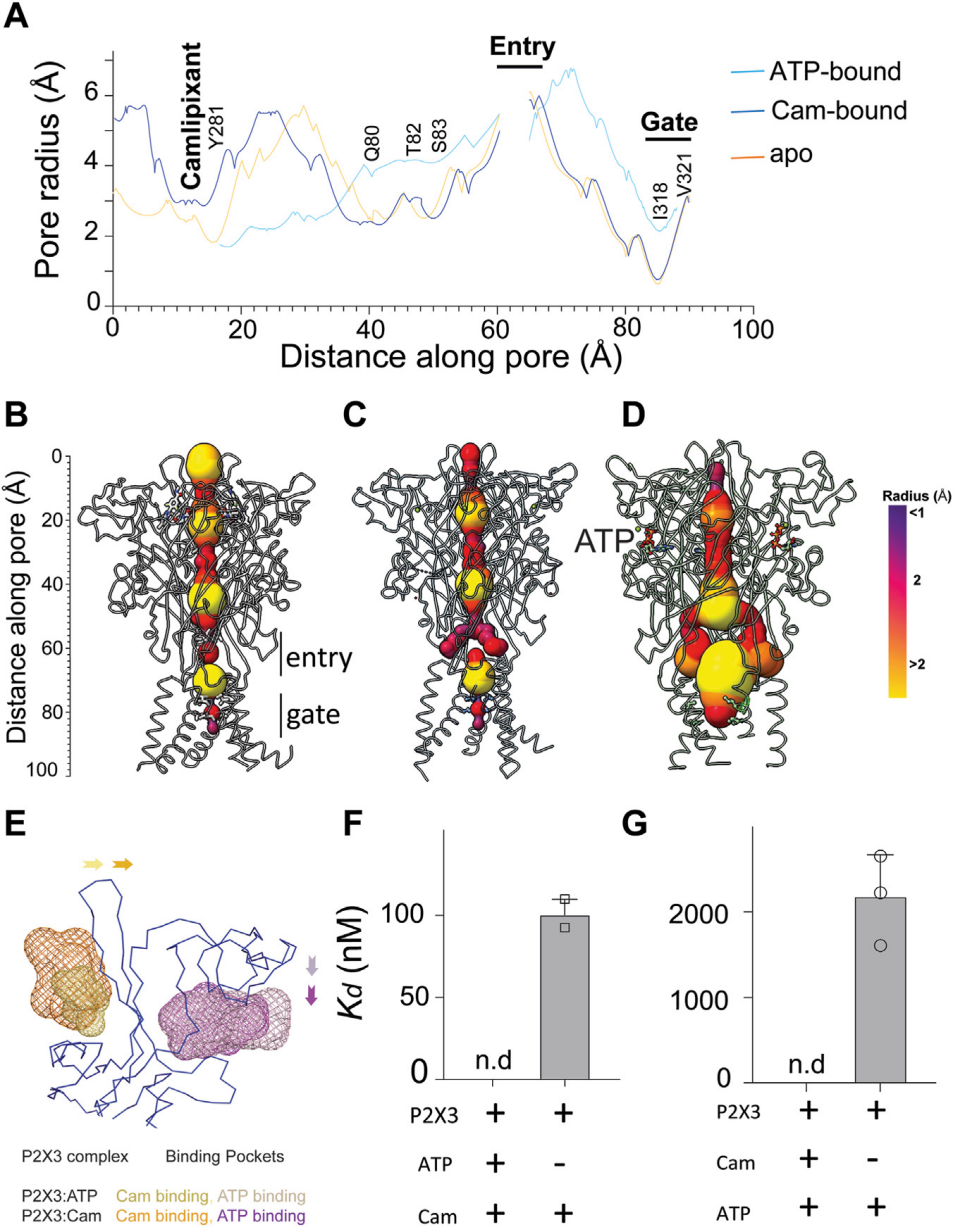
**The turret of the P2X3 receptor opened, but the ion entry and gate remained closed in camlipixant (Cam)-bound state.***A*, the channels formed in Cam-bound, apo, and ATP-bound states of P2X3 are represented by *line diagrams*. Residues lining the selectivity filter and surrounding the gate are highlighted. Cam and Y281 residue involving in turret formation. *B*–*D*, surface representations of the channels from Cam-bound, apo, and ATP-bound state. *E*, the alterations in the ATP-binding and drug-binding pockets between the ATP-bound and Cam-bound states. A conformational change upon Cam binding, evidenced by the enlargement of the Cam-binding site and the narrowing down of the ATP-binding site (indicated by *arrows*). The binding of either ATP or CaM to the P2X3 receptor mutually excludes the subsequent binding of the other, as demonstrated by ITC (*F*) or MST (*G*). The error bars represent the standard deviation, with n = 2 for ITC and n = 3 for MST. ITC, isothermal titration calorimetry; MST, microscale thermophoresis.

To elucidate the mechanism of camlipixant-bound inactivation of the P2X3 receptor, we determined the cryo-EM structure of the P2X3–ATP complex (Figs. S11 and S12, and Table S1). The entire channel, approximately 85 Å in length, was divided into two parts: the turret and the transmembrane channel separated by ion entry (Fig. 3*A*). The ion entry and gate were widened in the ATP-bound state but closed in the camlipixant-bound state (Fig. 3*A*). Upon camlipixant binding, P2X3 underwent conformational changes, adopting a closed state as indicated by the constriction of the channel gate formed by transmembrane helices at residues I318 and V321 (Fig. 3, *A–D*). The upper body domain experienced a shift, moving residues primarily from the β-strands (β3, β4, β13, and β14) backward. This transition enlarged the camlipixant-binding pocket, increasing its volume, from approximately 241 Å³ in ATP-bound state to 513 Å in the apo state, and finally to 1203 Å^3^ in the camlipixant-bound state (Figs. 3*E* and S13, *A* and *B*, and Videos S1 and S2). Meanwhile, the loop of the ATP-binding pocket shifted closer, reducing its volume from 1161 Å^3^ in apo and ATP-bound states to 573 Å^3^ (Figs. 3*E* and S13, *A–C*, and Videos S1 and S2). This alteration prevents ATP-mediated receptor activation of P2X3 upon binding to camlipixant.

In addition, upon camlipixant binding, the transmembrane helices moved closer to the gate (Videos S1 and S2). These movements are coupled with widening of the lower body domain, albeit to a degree where the transmembrane helices remain closed. Interestingly, we also observed changes in the turret volume, which is closed in the ATP-bound state, partially open in the apo, and fully open in the camlipixant-bound state, resulting from conformational changes in residues Q80, T82, and S83 (Fig. 3, *A–D*). Camlipixant binding disrupts conformational changes in the upper body domain, including residue Y281 (Fig. 3, *A–D*), causing insufficient movement of the pore-lining transmembrane helices to simultaneously open the channel. Such conformational changes are required to prevent ATP binding and stabilize channel closure.

Our structural analysis is substantiated by biochemical experiments showing that camlipixant binds noncompetitively to P2X3 and allosterically inhibits ATP-induced receptor activity. No binding of camlipixant to P2X3–ATP was observed, whereas camlipixant binds to P2X3 with no ATP bound with a *K*_*d*_ of 100 nM using isothermal titration calorimetry (ITC) (Figs. 3*F* and S14). These data indicate that camlipixant binds to pockets exposed to the ATP-unbound state (also called the resting state). Intriguingly, we also observed that the camlipixant-bound receptor was incapable of binding to ATP–Mg^2+^, suggesting that camlipixant occluded the ATP-binding site (Fig. 3*G*). ATP binds to P2X3 with a *K*_*d*_ of 2μM, as measured by microscale thermophoresis (MST) (Figs. 3*G* and S15). Notably, literature reports vary regarding this binding affinity, ranging from nanomolar to micromolar (17).

## Discussion

The specific localization of P2X3 receptors in primary sensory neurons plays a crucial role in modulating synaptic transmission in various tissues and organs. Their significance is particularly pronounced in cough reflex, pain perception, and bladder function (18). The development of drugs that target P2X3 receptors is a crucial pursuit in clinical research. Nonetheless, achieving subtype selectivity is imperative to minimize the toxic side effects and enhance the potential for successful drug development. Despite the significant pharmaceutical interest in targeting P2X3, no drug has received Food and Drug Administration approval to date. Therefore, chronic cough, chronic obstructive pulmonary disorder, and bladder disorders remain unmet clinical needs.

There has been extensive interest in the search for molecules that act as selective antagonists of P2X3 over the P2X2/3 and P2X receptors. Among the numerous new antagonists targeting P2X3 that have emerged in recent years, camlipixant is currently undergoing phase III clinical trials (NCT05599191). The efficacy and selectivity of the camlipixant were evaluated across various P2X receptors using cell-based calcium mobilization assays, following ATP activation (8). Camlipixant exhibited potent and noncompetitive antagonistic effects on human P2X3 receptors, with an IC_50_ of approximately 20 nM (8). Conversely, camlipixant did not demonstrate significant antagonistic activity against hP2X_1–7_ receptors, with EC_50_ values in the micromolar range (8).

**Figure 4:**
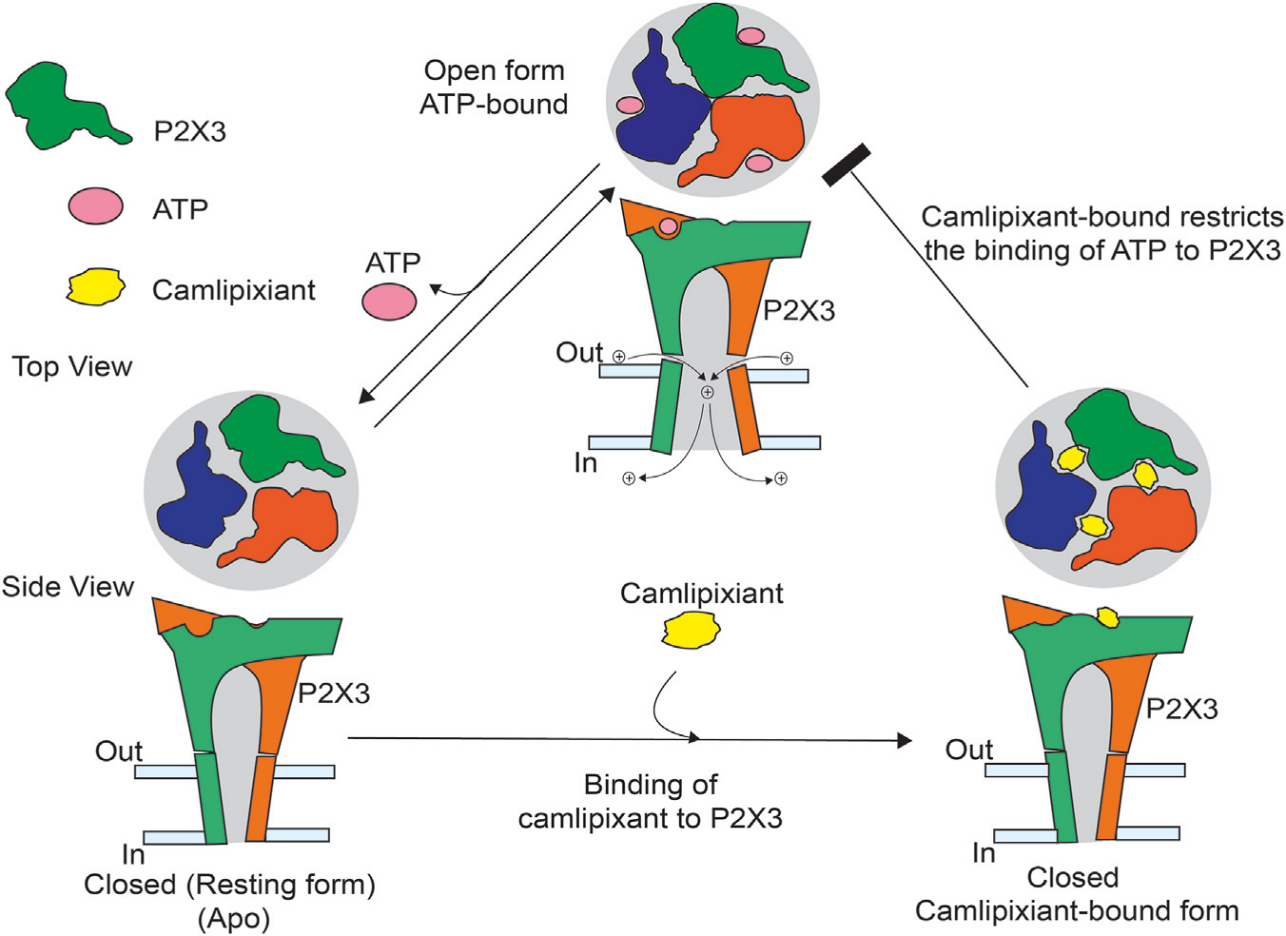
**Mechanisms underlying camlipixant-bound P2X3 inhibition.** Schematic representations of the P2X3 receptor, viewed from the *top* and *side*, display distinct subunits in different colors. As the channel is activated by binding ATP, the drug-binding pocket narrows, and the ion entry gate opens. Upon ATP dissociation, a conformational change occurs in the receptor, enlarging the drug-binding pocket to facilitate drug binding; ion entry and gate closed. Camlipixant, selective to P2X3, stabilizes the closed conformation by obstructing the movement of intersubunit cavities, leading to the closure of the ATP-binding site.

The cryo-EM structure of a P2X3–camlipixant complex identified a cryptic drug-binding site for camlipixant in the P2X3 receptor. Through structural analyses and biochemical and biophysical experiments, we found that camlipixant binds to accessible pockets present in the resting state, rather than solely to sites exposed when the channel is open, such as those deeper within the upper body domain. Camlipixant binding induces conformational changes in the extracellular domain machinery that block the ATP-binding site and stabilize the closed ion entry gate (Fig. 4). The gate's radius diminishes to 0.7 Å (Fig. 3, *A* and *B*). This alteration signifies the obstruction of cation transfer, encompassing Ca^2+^, Na^+^, and K^+^ with ionic radii of 0.99, 0.95, and 1.33 Å, suggesting potential implications for neuronal signaling. The druggable site is situated within the interface created by β-strands 3, 4, and 13, 14 in the upper body region of P2X receptors. Sequence identity is moderately conserved across the P2X family (Table S2). The binding site provides significant selectivity for the P2X3 homotrimer rather than the P2X2/3 heterotrimer, which provides a molecular explanation for the physiological observation of not dampening the taste response, while still reducing the cough response.

## Experimental procedures

### P2X3 construction, expression, and purification

The gene encoding P2X3 in *Canis lupus* sp. (P2X3) was cloned into the pElanco3 vector with the deletion of five first residues in the N-terminal region and the 33 last residues in the C-terminal region, as previously described in human P2X3 (10). The gene construct expressed the 12His-eGFP-GGS-(thrombin)-GGS-protein-GGS-(HRV3c)-Rho1E tag with codon optimization in the human embryonic kidney cell line. To generate the P2X3-Expi293F GnTI^-^ suspension cell line (ThermoFisher), the pElanco3-eGFP-P2X3 Transposon and PiggyBac Transposase plasmids were transfected at a 1:20 ratio of transposase–transposon using a Neon Electroporation System. Briefly, 5 × 10^7^ cells were transfected with 10 μg of total DNA using the Neon 100 μl tip 6-well plate protocol. The cells were electroporated with two pulses of 1100 V each, with a pulse width of 20 ms, and then transferred into 2 ml of prewarmed Gibco Expi293 Expression Media (catalog no.: A1435101) in a low-attachment 6-well plate and incubated at 37 °C in a humidified 8% CO_2_ incubator for 1 day. After 24 h, GFP expression was verified using fluorescence microscopy, and 2 μg/ml puromycin (ThermoFisher; catalog no.: A1113803) was added to initiate cell selection. After 7 days of puromycin selection, the cells were expanded in Corning Erlenmeyer flasks in Expi293 medium supplemented with 1 μg/ml puromycin.

P2X3 was expressed and purified from a stable cell line P2X3-Expi293. The cells were disrupted using a sonicator, operating at 45% amplification with a pulse of 10 s on and 10 s off, in buffer A (20 mM Tris–HCl [pH 7.6], 150 mM NaCl, 0.1 mM body domain Tris(2-carboxyethyl)phosphine [TCEP], and 5% glycerol) supplemented with an EDTA-free protease inhibitor cocktail (ThermoFisher; catalog no.: PIA32955). Undisrupted cells were removed by centrifugation at 4000 rpm for 10 min at 4 °C. The resulting suspension, containing disrupted cells and membranes, was harvested and subjected to ultracentrifugation at 40,000 rpm for 1 h at 4 °C. The membrane-containing pellet was homogenized using a homogenizer in buffer B (20 mM Tris–HCl [pH 7.6], 150 mM NaCl, 0.1 mM TCEP, and 2% DDM) supplemented with a protease inhibitor cocktail. After rotating the homogenized solution for 2 h at 4 °C, it was subjected to ultracentrifugation. The obtained soluble fraction was applied to Talon beads (Takara; catalog no.: 635503) and incubated for 6 h at 4 °C. Subsequently, the beads were washed with buffer C (20 mM Mes [pH 6.5], 150 mM NaCl, 0.1 mM TCEP, and 0.2% DDM) before treatment with thrombin and HRV3C protease in the same buffer for 6 h at 4 °C. The P2X3 fraction was then harvested and subjected to size-exclusion chromatography (Superdex 200 10/300 column) in buffer D (20 mM Hepes [pH 7.5], 100 mM NaCl, and 0.08% DDM). Peak fractions containing P2X3 were pooled and analyzed by SDS-PAGE. Monodisperse fractions of P2X3 were used for cryo-EM grid preparation.

### Camlipixant-bound P2X3–peptidisc reconstitution

To completely dissociate ATP from the P2X3 receptor, the sample was subjected to dialysis in a buffer containing 20 mM Tris–HCl (pH 7.6), 1000 mM NaCl, and 0.08% DDM supplemented with protease inhibitor for 7 days. The buffer was refreshed daily before returning it to buffer D. The peptide sequence (FAEKFKEAIKDYFAKFWDPAAEKLKEAIKDYFAKLW) was chemically synthesized using solid-phase peptide synthesis (GenScript) to achieve a purity of >85%. The peptide was dissolved in buffer E (20 mM Hepes [pH 7.5] and 100 mM NaCl) to a final concentration of 1 mM. To reconstitute P2X3 into the peptidisc, a sample of purified P2X3 was incubated on ice for 30 min with excess peptidisc solution at a 1:20 M ratio. The P2X3–peptidisc complex was separated from the free peptidisc by size-exclusion chromatography. The peak fraction containing P2X3–peptidisc was analyzed by SDS-PAGE. Subsequently, the P2X3–peptidisc complex was incubated with camlipixant (Pharmaron) at a molar ratio of 1:5 for 30 min on ice before preparing cryo-EM grids.

### Cryo-EM grid preparation and data acquisition

A monodisperse fraction of P2X3 was used for grid preparation. Holey carbon gold Quantifoil grids (R1.2/1.3 mm, 300 mesh) were glow-discharged for 60 s at 25 mA, holding for an additional 10 s. Subsequently, 3.0 μl of the sample was applied to the grids and blotted for 2 s at 4 °C and 100% humidity, followed by vitrification by plunging into liquid ethane cooled by liquid nitrogen using a Vitrobot Mark IV (ThermoFisher). Micrographs were captured using a Titan Krios (ThermoFisher) operating at 300 kV with a nominal magnification of 105,000× for all datasets. The micrographs were obtained using a nominal defocus value of 0.8 to 2.0 μm. A calibrated pixel size of 0.4125 Å was used for processing. Videos were recorded using Leginon 3.6 (National Resource for Automated Molecular Microscopy) (19) at a dose rate of 28.56 e−/Å2/s with a total exposure time of 1.80 s, accumulating a dose of 56.8 e−/Å^2^.

### Electron microscopy data processing

Image processing and structural determination involve several steps. First, motion correction and summation of the images were performed using MotionCor2 (National Center for Macromolecular Imaging) (20). Contrast transfer function parameters for each non–dose-weighted micrograph were estimated as defocus values using Gctf (21). Particle picking was performed using the DoG Picker (22), followed by initial reference-free 2D classification using CryoSPARC (Structural Biology Software Solutions, Inc), version 4.2.0 (23). Representative 2D class averages were selected, and further particle cleanup was performed with multiple rounds of 2D classification. Subsequently, initial *ab initio* reconstruction was performed.

These refined particles were further subjected to nonuniform refinement (24), which generated a map with the indicated global resolution at a Fourier shell correlation of 0.143. The density map was refined by local refinement with a tight mask covering only the protein region, whereas the surrounding peptidisc was manually removed using UCSF Chimera (UCSF) (version 1.16) (25). The final volume map was calculated with C3 symmetry, and the local resolution was estimated using PHENIX (PHENIX Consortium) (26) and displayed using PyMol (Schrödinger, LLC) (27) and Chimera.

### Model building and structure determination

The initial apo state model of P2X3 was constructed in Coot (MRC Laboratory of Molecular Biology), version 0.8.9 (28), using the AlphaFold (29) structure as a reference. The individual monomer P2X3 structures were fitted to cryo-EM maps using Chimera, followed by successive manual adjustments and rebuilding iterations using Coot. Throughout the model-building process, manual refinements were performed based on the map quality in Coot, followed by real-space refinement in PHENIX (26).

In both complex structures, residues ranging from 5 to 21 were missing from the models because of the absence of corresponding density in the maps. The geometric quality of the atomic models was evaluated using MolProbity (University of North Carolina) (30). The detailed refinement statistics are provided in Table S1. The pore-lining surfaces and channels within the receptor were calculated using HOLE (31) and MOLE (32), respectively.

### Preparation of protein and ligand structures and molecular docking

For comparative analysis, three protein structures were selected: P2X7:GW791343 from *Ailuropoda melanoleuca* (PDB ID: 5U1Y), zebrafish P2X4:BX430 (PDB ID: 8JV5), and P2X3–camlipixant (in-house). In addition, a homology model of the P2X2/3 heterotrimer (one P2X2 and two P2X3 subunits) was included. Each protein structure exists in a trimeric form with three identical ligands bound to each monomer.

All protein structures were prepared using default parameters *via* the protein preparation workflow of Schrödinger Suite, version 2023-4. Briefly, water molecules were removed to maintain homogeneity in the protein-binding pockets. Hydrogen atoms were added at pH 7.4, missing side chains were filled, and the hydrogen-bonding network was optimized using PROPKA (University of Copenhagen) (33). Disulfide bonds were created where possible, and proteins were minimized to achieve an RMSD gradient of 0.30 Å compared with the starting structure using the OPLS4 force field.

The prepared structures were used to generate protein grids. Active site grids were generated around the ligands present in chain A of each protein. For the P2X2/3 heterotrimer structure, an active-site grid was generated around the modeled ligand present in P2X2 (chain A) with either chain B or C of P2X3. The grid box size of 27.1 Å^3^ (*outer box*) and 10.0 Å^3^ (*inner box*) was defined as the center of mass of the respective ligand. The generated grids were used for the molecular docking of the ligands. Ligands were separated and prepared using the LigPrep module, where ionization states were generated at pH 7.0 ± 2.0, using Epik (Schrödinger, LLC) (34), retaining input chirality. Molecular docking calculations were performed in extra precision (XP) mode with default parameters using the Glide module. Docking of all three ligands was performed in all four structures as a matrix, and the extra precision scores were recorded. Subsequently, molecular mechanics with generalized born surface area binding free energy was calculated for each protein–ligand complex using the Prime MMGBSA module.

### MD simulation

The docked complexes served as starting structures for MD simulations conducted using the Desmond simulation package (Schrödinger Suite). Prior to MD simulations, Desmond's default system relaxation protocol was used for equilibration. This included a series of stages: a 100 ps Brownian dynamics NVT simulation at 10 K with constraints on the heavy atoms of the protein, followed by 12 ps NVT simulations at 10 K and 12 ps NPT simulations at 10 K, both with restrictions on solute heavy atoms. The final relaxation stage involved increasing the temperature from 10 K to 300 K in 12 ps under NPT ensemble conditions, followed by a 24 ps NPT ensemble simulation at 300 K with no restraints on any atom. Two complexes, P2X3–camlipixant and P2X2/3–camlipixant, were simulated for 100 ns, with a recording interval of 100 ps, utilizing OPLS4 force-field parameters.

### ITC and MST measurements

ITC measurements were conducted at 20 °C using a NANO ITC system (TA Instruments) to assess the drug binding affinity of P2X3. Prior to use, all protein camlipixant solutions were equilibrated in the same degassed buffer D. ITC cells were loaded with 250 μl of the P2X3 receptor (5 μM) and titrated against the drug compound (20 μM). Titration was performed by injecting 0.1 μl of substrate into the protein, followed by 19 injections of 0.25 μl each. The stirring rate was maintained at 200 revolutions per minute. For the control experiments, the drug was injected into the buffer alone. ITC measurements were conducted two times using samples from the same batch of purified proteins (technical repeats).

MST measurements were conducted using the Monolith NT.115 system (NanoTemper Technologies). Briefly, fluorescein-labeled P2X3 was prepared in buffer D according to the manufacturer's instructions (Red Tris second generation dye). A 2-fold dilution series of camlipixant was then prepared, resulting in final concentrations ranging from 20 μM to 0.61 nM. The samples were then incubated on ice for at least 30 min. After incubation, samples were loaded into premium-coated capillaries and subjected to MST analysis. The results were analyzed using TJump analysis. The dissociation constant was determined by fitting with a dose–response model provided by the manufacturer. MST experiments were conducted three times using samples from the same batch of purified proteins (technical repeats). ITC and MST data are plotted using GraphPad Prism and presented as the mean ± standard deviation.

### Intracellular calcium influx assay

The assay was performed using the Fluo-8 Calcium Flux Assay Kit (Abcam; catalog no.: ab112129), following the manufacturer's instructions. Cells were centrifuged from the culture medium and resuspended in an equal amount of Fluo-8 dye-loading solution, along with Hank’s balanced salt solution supplemented with calcium (Gibco). A suspension containing 2.0–2.5 × 10^5^ P2X3-Expi293 cells per well was prepared in a 96-well poly-d-lysine plate. The dye-loading plate was incubated in a cell incubator for 30 min at 37 °C with 8% CO_2_. ATP–Mg^2+^ was used in the range of 0 to 2 mM, and camlipixant was used in the range of 0 to 2 mM in the assay. These compounds were dispensed directly onto cell plates, and the data were collected simultaneously. A fluorescence-based assay was used to detect intracellular calcium mobilization in cells at excitation/emission wavelengths of 490/525 nm, using a microplate reader G5 (SpectraMax). The readings obtained from the blank standard were used as negative controls. This value was subtracted from the readings of other standards to obtain the baseline-corrected values. The experiments were repeated three times with the cells from the same batch.

## Data availability

Three-dimensional cryo-EM density maps and coordinates for camlipixant-bound P2X3 (accession codes: EMD-44771 and 9BPC) and ATP-bound P2X3 (accession codes: EMD-44772 and 9BPD) were deposited in the PDB. An overall map was used to refine the ATP-bound P2X3 structure. In addition, focused refinements were conducted on both the extracellular domain (excluding the transmembrane domain) and transmembrane domain (excluding the extracellular domain) to enhance the visualization of specific features present in the overall map. Although these refined maps aided in model building, they were not utilized for refinement in PHENIX.

## Supporting information

This article contains supporting information.

## Conflict of interest

P. P. N., S. S., I. H., S. A., and J. S. work for ELANCO. All the other authors declare that they have no conflicts of interest with the contents of this article.

## Supporting information

Supplemental Figs. S1–S15, Tables S1–S3, and Videos S1 and S2
