# Supplementary material for: Mechanistic insights into the selective targeting of P2X3 receptor by camlipixant antagonist
Source: J Biol Chem. 2024 Dec 18;301(1):108109. doi: 10.1016/j.jbc.2024.108109 (PMC11783113; doi:10.1016/j.jbc.2024.108109)
Supplement: Supplemental Figs. S1–S15, Tables S1–S3, and Videos S1 and S2 [file mmc1.docx]

Supporting Information.

**Mechanistic insights into the selective targeting of P2X3 receptor by camlipixant antagonist**

Trung Thach^1*^, KanagaVijayan Dhanabalan^1^, Prajwal Prabhakarrao Nandekar^1^, Seth Stauffer^2^, Iring Heisler^2^, Sarah Alvarado^2^, Jonathan Snyder^2^, Ramaswamy Subramanian^1,3*^

^1^Department of Biological Sciences, Purdue University, West Lafayette, IN-47907, USA

^2^Elanco Animal Health, 2500 Innovation Way, Greenfield, IN-46140, USA

^3^Weldon School of Biomedical Engineering, Purdue University, West Lafayette, IN-47907, USA.

*Corresponding authors: [subram68@purdue.edu](mailto:subram68@purdue.edu) and [ttthach@purdue.edu](mailto:ttthach@purdue.edu)

1. SUPPLEMENTARY TaBLES

Table S1. Cryo-EM data collection, refinement, validation and statistics

Table S2. Residues within the camlipixant binding pocket and sequence alignment across the P2X receptor family

Table S3. Molecular dynamics simulations were conducted across various P2X members using different ligands

1. Supplementary Figures

Fig. S1. The generation process of the Expi293F GnTI-stable

Fig. S2. The Expi293F GnTI^-^ stable cell line expressing functional P2X3 receptor

Fig. S3. The impact of peptidisc in cryo-EM P2X3:camlipixant structure determination

Fig. S4. The cryo-EM data processing pipeline for P2X3:camlipixant reconstituted into peptidisc

Fig. S5. Cryo-EM structures of the camlipixant-bound P2X3 receptor, reconstituted into either DDM or peptidisc

Fig. S6. The binding pocket for camlipixant in the P2X3 receptor exhibits moderate conservation among P2X receptors

Fig. S7. Sequence alignment of *Canine lupus* P2X3 (cP2X3) and Human P2X3 (hP2X3)

Fig. S8. The sequence alignment of cP2X3 and hP2X2 isoforms

Fig. S9. Molecular dynamics simulations of camlipixant-bound P2X2/3 receptor

Fig. S10. The Cam binding site appears across P2X receptors

Fig. S11. The cryo-EM data processing pipeline for P2X3:ATP complex

Fig. S12. The cryo-EM structure of the P2X3:ATP complex.

Fig. S13. The drug-binding pocket enlarges in the camlipixant-bound P2X3 receptor

Fig. S14. The binding affinity of camlipixant to P2X3 was determined using ITC

Fig. S15. The binding affinity of ATP to P2X3 was determined using MST

1. Supplementary Videos

Video S1. Conformational changes in P2X3 upon binding to camlipixant compared to its apo structure

Video S2. Conformational changes in P2X3 upon binding to camlipixant compared to its ATP-binding structure

Table S1. Cryo-EM data collection, refinement, validation and statistics.

| **Structures** | **P2X3:ATP in DDM**  PDB-ID, 9BPD  EMD-44772 | **P2X3:Cam in DDM** | **P2X3:Cam in peptidisc**  PDB-ID, 9BPC  EMD-44771 | |
| --- | --- | --- | --- | --- |
| **Data collection and processing** |  | | | |
| Magnification | 105,000 | 105,000 | 105,000 |  |
| Voltage (kV) | 300 | 300 | 300 |  |
| Electron exposure ((e–/Å2) | 56.8 | 56.8 | 56.8 |  |
| Defocus range ((μm) | 0.8-2.0 | 0.8-2.0 | 0.8-2.0 |  |
| Raw pixel size (Å) | 0.411 | 0.411 | 0.411 |  |
| Symmetry imposed | C3 | C3 | C3 |  |
| Number of initial particle images | 87,363 | 561,446 | 200,982 |  |
| Number of final particle images | 44,237 | 373,772 | 40,142 |  |
| Map resolution (Å) | 3.63 | 2.93 | 3.44 |  |
| FSC threshold | 0.143 | 0.143 | 0.143 |  |
| Map resolution range (Å) | 3.0-5.0 | 2.5-5.0 | 3.0-5.0 |  |
|  |  |  |  |  |
| **Refinement** |  |  |  |  |
| Initial model used | Alphafold model | Apo model | Apo model |  |
| Model resolution (Å) | N/A | 3.6 | 3.6 |  |
| *FSC threshold* | 0.5 | 0.5 | 0.5 |  |
| *Model resolution range* (Å) | N/A | 3.0-50 | 3.0-50 |  |
| *Map sharpening B factor* (Å^2^) | -100 |  | -90 |  |
| *Model composition* |  |  |  |  |
| *Non-hydrogen atoms* | 7551 |  | 7362 |  |
| *Protein residues* | 918 |  | 927 |  |
| *Ligand: Mg* | 3 |  |  |  |
| *ATP* | 3 |  |  |  |
| *Camlipixant* |  |  | 3 |  |
| NAG | 15 |  | 9 |  |
| B factors *(*Å^2^) |  |  |  |  |
| Protein | 160.45 |  | 85.50 |  |
| Ligand | 168.09 |  | 80.68 |  |
| RMSD values |  |  |  |  |
| Bond lengths (Å) | 0.003 |  | 0.004 |  |
| Bond angles (^o^) | 0.634 |  | 0.612 |  |
| Validation |  |  |  |  |
| Molprobity score | 2.63 |  | 2.09 |  |
| Clash score | 13.18 |  | 8.51 |  |
| Poor rotamer (%) | 0.38 |  | 1.20 |  |
| Ramachandran plot (%) |  |  |  |  |
| Favored | 87.50 |  | 89.04 |  |
| Allowed | 12.17 |  | 10.96 |  |
| Outliers | 0.33 |  | 0 |  |

Table S2. Residues within the camlipixant binding pocket and sequence alignment across the P2X receptor family. Conserved residues are highlighted in bold letters. -, deletion/insertion.

| cP2X3 | HUMAN | | | | | |
| --- | --- | --- | --- | --- | --- | --- |
|  | P2X3 | P2X2 | P2X4 | P2X5 | P2X6 | P2X7 |
| Y65 | **Y70** | L/S | T75 | T76 | T59 | V64 |
| R68 | **R73** | H/K | **R83** | **R84** | **R65** | S78 |
| M70 | **M75** | G/W | W85 | W86 | W67 | F80 |
| I88 | **I93** | - | V102 | V104 | L82 | V106 |
| M91 | **M96** | - | V105 | L107 | F106 | F111 |
| M160 | **M165** | G | F179 | L182 | L137 | L184 |
| F277 | **F282** | F | **F297** | **F299** | T245 | Y295 |
| Y280 | **Y285** | Y | **Y300** | **Y302** | H248 | **Y305** |
| E288 | **E293** | T | **E308** | **E310** | **E256** | **E313** |
| L293 | **L298** | I | I313 | M315 | **L261** | I318 |

Table S3. Molecular dynamics simulations were conducted across various P2X members using different ligands. The simulations yielded MMGBSA binding free energy values (kcal/mol), depicted in a heatmap where lighter shades (white) indicate highest energy levels, while darker shades (dark red) indicate lowest energy levels. P2X4 (PDB-ID, 8JV5), P2X7 (PDB-ID, 5U1Y).

| **Protein**  **Ligand** | **P2X7-5U1Y** | **P2X4-8JV5** | **P2X3** | **P2X3-P2X2 Interface** | **P2X2-P2X3 Interface** |
| --- | --- | --- | --- | --- | --- |
| GW791343 | -67.38 ± 6.04 | -55.36 ± 3.98 | -50.72 ± 3.95 | -52.57 ± 3.83 | -55.27 ± 3.95 |
| BX430 | -56.49 ± 5.99 | -58.54 ± 2.99 | -36.69 ± 4.91 | -54.66 ± 4.61 | -58.69 ± 6.08 |
| Camlipixant | -61.92 ± 3.72 | -72.54 ± 4.32 | -74.53 ± 3.32 | -68.25 ± 4.64 | -47.58 ± 4.69 |


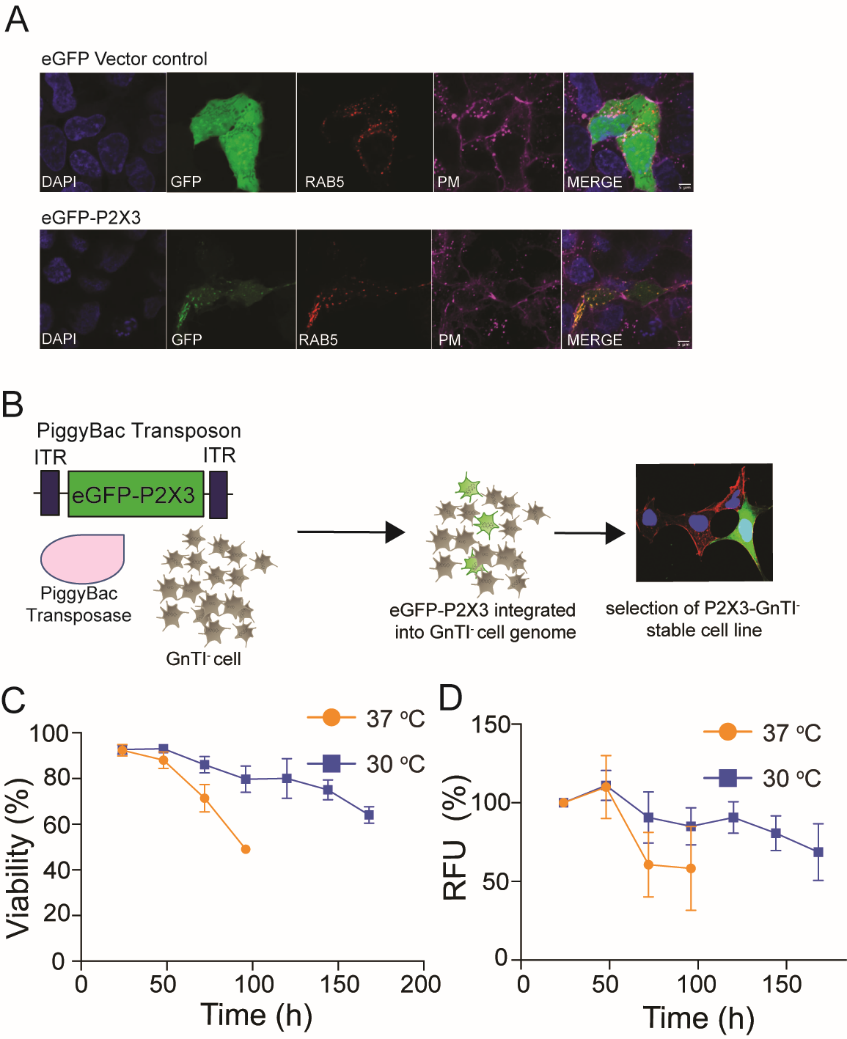


**Fig. S1. The generation of Expi293F GnTI- stable cell line expressing eGFP-P2X3.** (A) eGFP expression was monitored using fluorescence confocal microscopy. Endosomes and the plasma membrane are identified through immunostaining using a Rab5 antibody and phalloidin staining, respectively. (B) The generation process of the Expi293F GnTI- stable pool through the piggyBac transposon system is outlined. ITR, inverted terminal repeat sequence. (C) The viability and relative fluorescence unit (RFU) of eGFP-P2X3 expression are depicted at 37 °C and 30 °C. Scale bar, 5 μm.


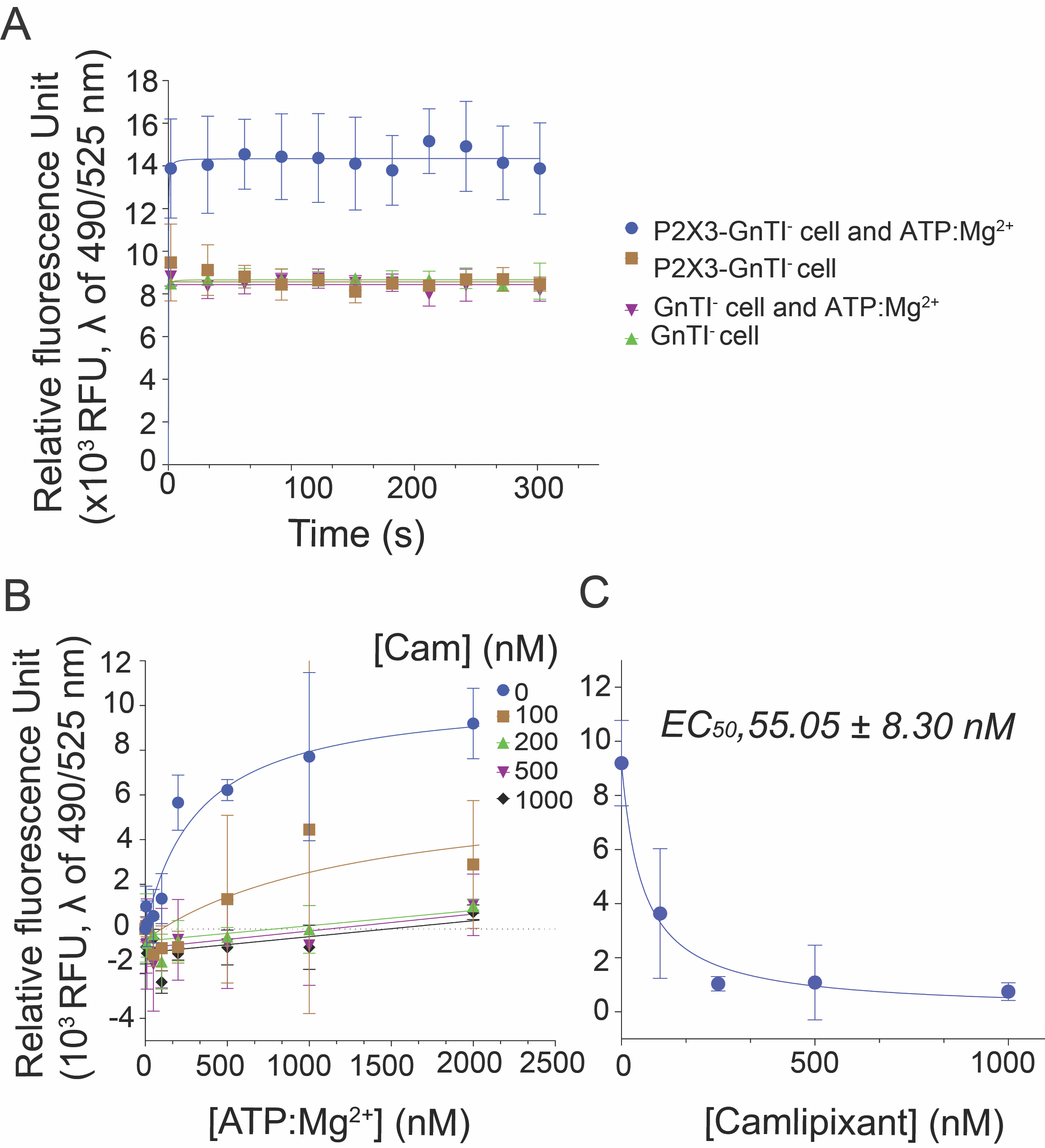


**Fig. S2. The Expi293F GnTI^-^ stable cell line expressing functional P2X3 receptor**. Intracellular Ca^2+^ signaling in GnTI^-^ and P2X3-GnTI^-^ stable cell lines are compared. The cells were loaded with the Ca^2+^-specific indicator Fluo-8 and stimulated with ATP:Mg^2+^. Error bars represent standard deviation, with n=3-6 for each test conducted. (B) Camlipixant's inhibitory effects on intracellular calcium flux by ATP:Mg^2+^ on the P2X3-GnTI^-^ cell line were measured. The change in intracellular Ca^2+^ was monitored for 5 minutes, with baseline measurements taken 1 minute before ATP:Mg^2+^ addition. There is a reduction in calcium uptake with increasing concentrations of camlipixant. (C) P2X3-GnTI^-^ cells were incubated with different concentrations of camlipixant for 30 minutes. Fluorescence was measured 5 min after addition of 2 μM ATP:Mg^2+^. Error bars represent standard deviation, with n=3 for each test conducted. n.d, not determined.


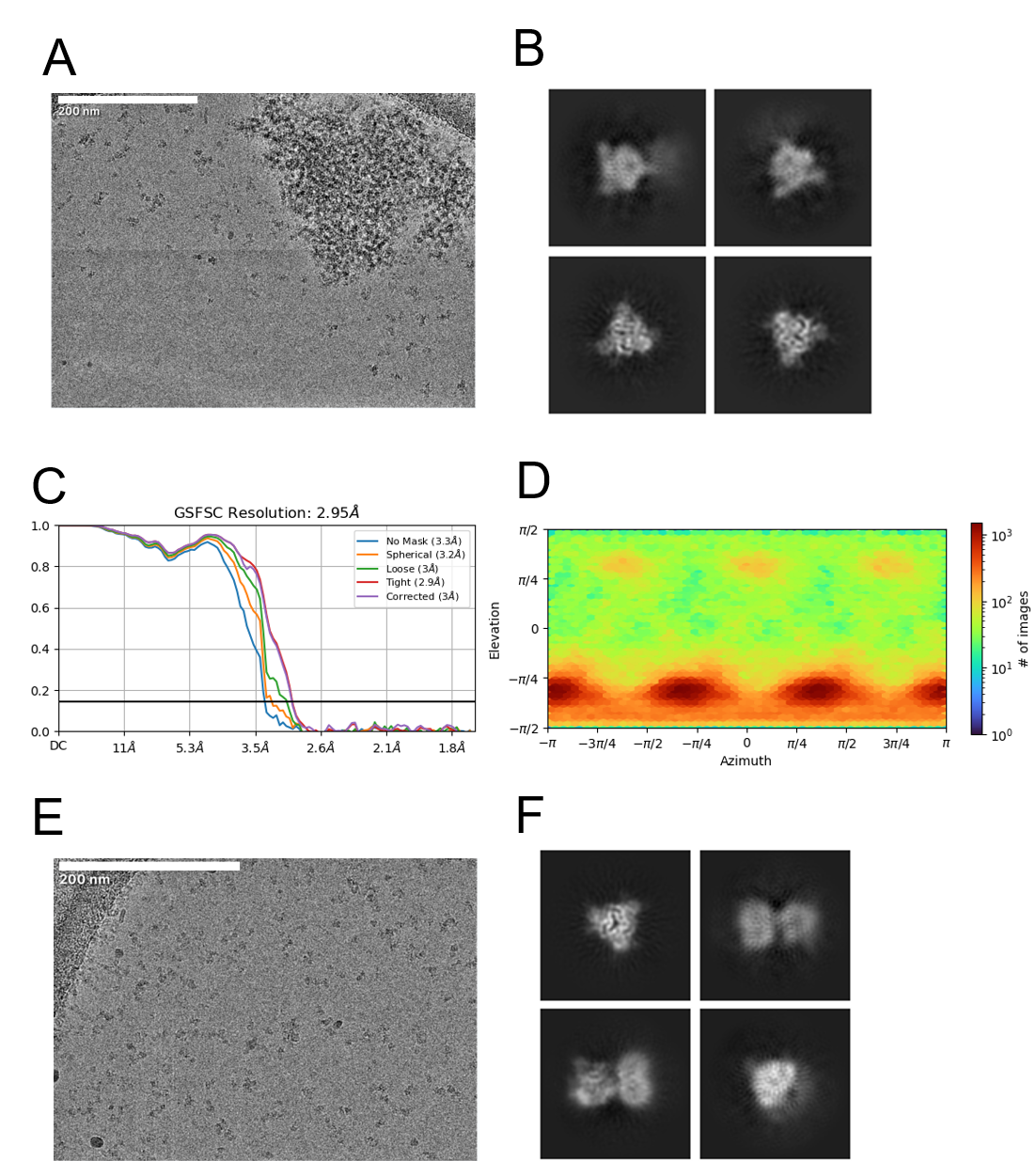


**Fig. S3. The impact of peptidisc in cryo-EM P2X3:camlipixant structure determination.** (A) Representative cryo-EM image of P2X3:camlipixant particles reconstituted into DDM. (B) 2D classification of P2X3:camlipixant reconstituted into DDM. The gold-standard Fourier shell correlation (FSC) curves for resolution estimation are displayed in (C), while the angular distribution of the particles used for the final map is illustrated in (D). (E) Representative cryo-EM image of P2X3:camlipixant particles reconstituted into peptidisc. (F) 2D classification of P2X3:camlipixant reconstituted into peptidisc is depicted.


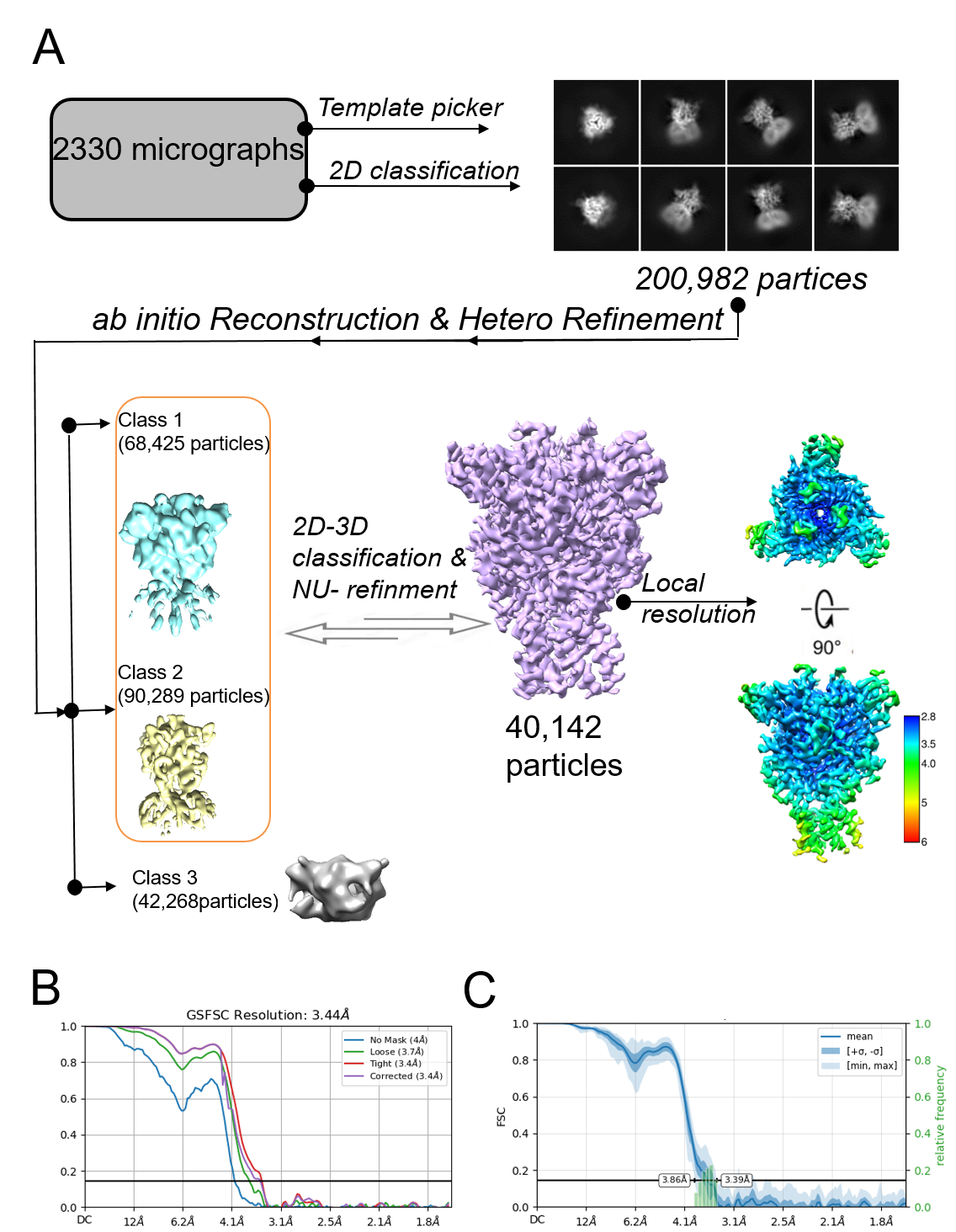


**Fig. S4. The cryo-EM data processing pipeline for P2X3:camlipixant reconstituted into peptidisc**. (A) An overview of the processing steps led to the generation of a cryo-EM map with an overall resolution of 3.44 Å, utilizing 20% of the initially exported particles. A local resolution map was calculated with cryoSPARC. This map indicates that the highest resolution (depicted in dark blue) correlates with the extracellular domain as observed in both side and top views from the extracellular side. The maps were visualized using UCSF ChimeraX. (B) Gold-standard Fourier shell correlation (FSC) curves were generated for resolution estimation. (C) The GSFSC curves, incorporating directional resolution, are plotted using cryoSPARC. The conical FSC (cFSC) is a Fourier Shell Correlation calculated between two half-maps, using a conical mask with a specified half-angle and axis in Fourier space. The variation in the cFSC curve, with directional resolutions differing by only about 0.5 Å, suggests that the structure exhibits a consistent directional resolution.


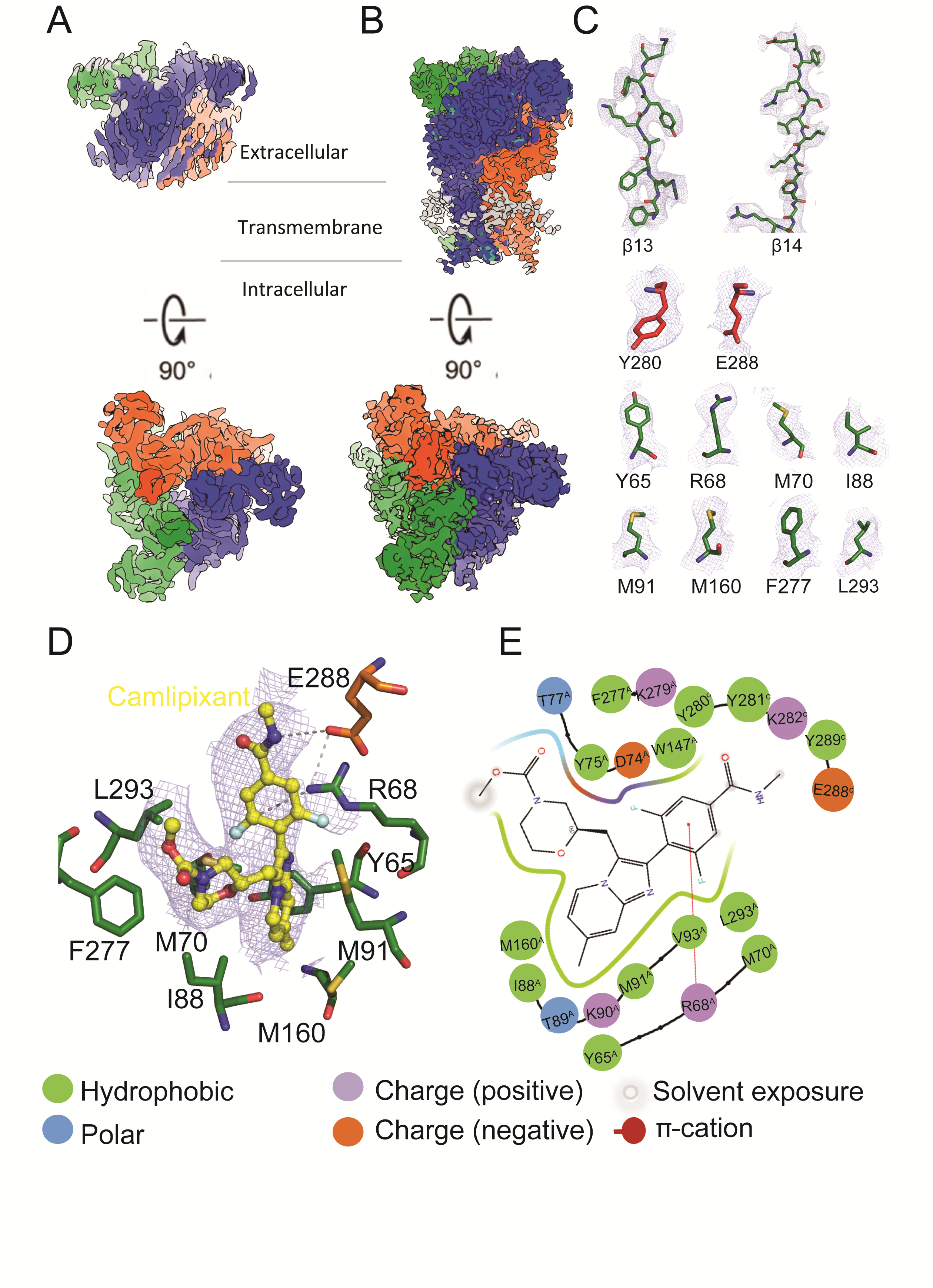


**Fig. S5. Cryo-EM structures of the camlipixant-bound P2X3 receptor, reconstituted into either DDM or peptidisc.** Representation of the cryo-EM map of the camlipixant-bound P2X3 receptor reconstituted into DDM (A) or peptidisc (B), shown inside view and top view. Each subunit color-coded is presented with the protomers of the trimer-colored blue, orange, and forest. (C) The representative residues involved in the β13, β14, and camlipixant binding pocket are shown as sticks, fitting well within the map. (D, E) A schematic diagram illustrating a close-up view of the interactions between camlipixant and P2X3. The drug is visualized as sticks and spheres. All cryo-EM maps are shown with contours set at 3.0 σ.


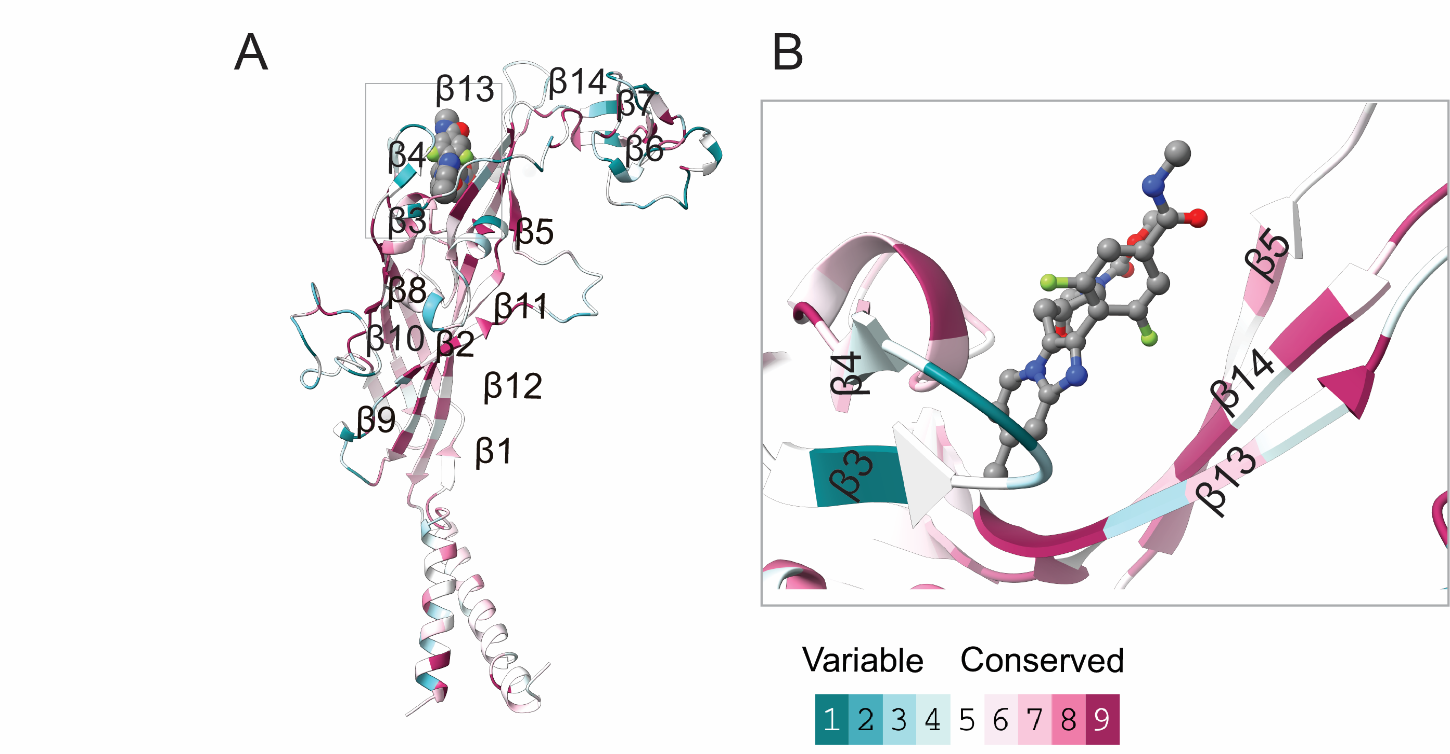


**Fig. S6. The binding pocket for camlipixant in the P2X3 receptor exhibits moderate conservation among P2X receptors. (**A) The evolutionary conservation of residue positions on the P2X3 structure is estimated based on phylogenetic connections among homologous sequences. The beta-sheet numbering is continuous from the N-terminus to the C-terminus. (B) A close-up view of the camlipixant binding site highlighting conserved residues.


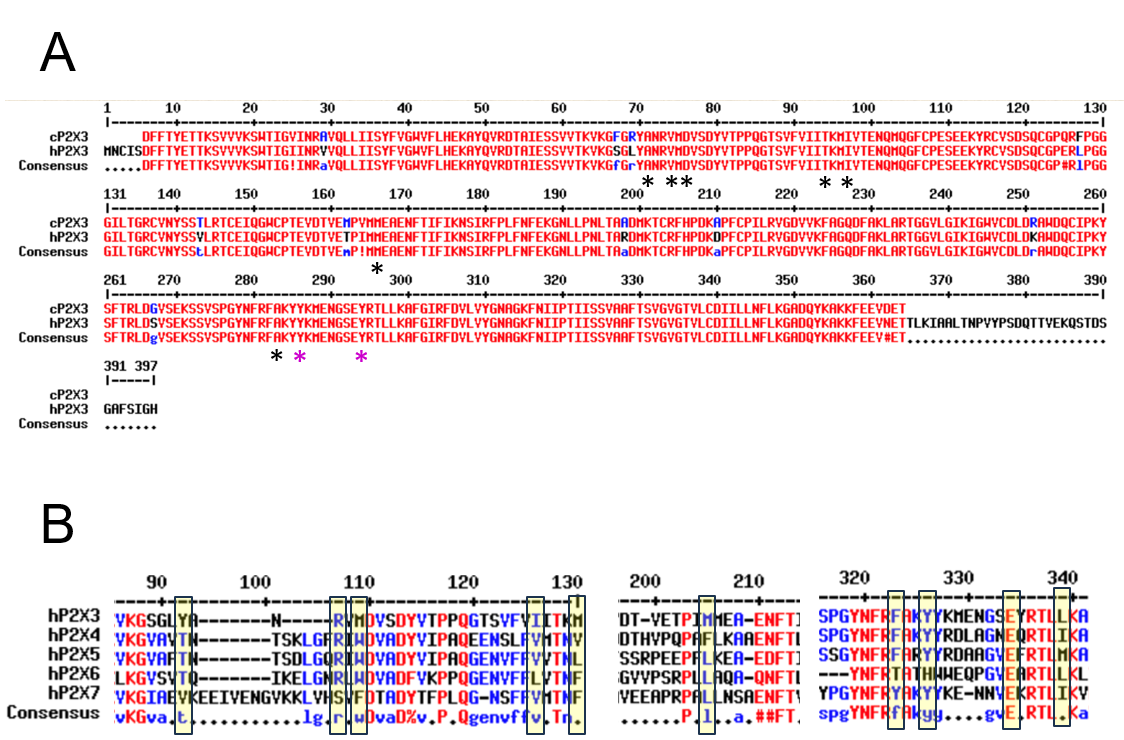


**Fig. S7. Sequence alignment of *Canine lupus* P2X3 (cP2X3) construct and human P2X3 (hP2X3).** (A) Black and pink stars indicate key residues in different subunits binding to camlipixant. Panel (B) shows the sequence alignment of hP2X3-7 receptors. Residues involved in the Cam binding pocket are highlighted within a yellow box. hP2X3 (NP_002550.2), hP2X4 (NP_001243725.1), hP2X5 (NP_001412012.1), hP2X6 (NP_001381624.1), hP2X7 (NP_002553.3).


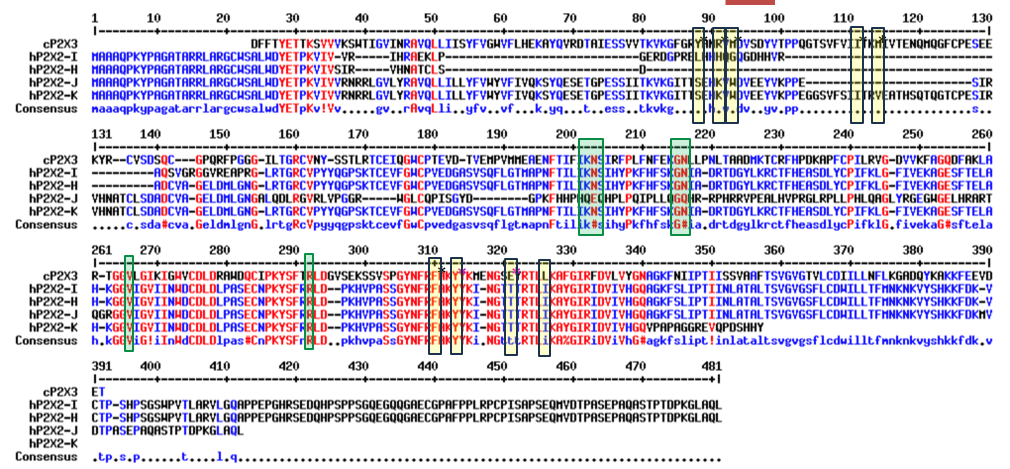


**Fig. S8. The sequence alignment of cP2X3 and hP2X2 isoforms.** Residues binding to camlipixant are highlighted within yellow boxes, while residues binding to gefapixant are highlighted within cyan boxes. hP2X2-I (NP_036358.2), hP2X2-H (NP_777361.1), hP2X2-J (NP_001269093.1), hP2X2-K (NP_001269094.1).


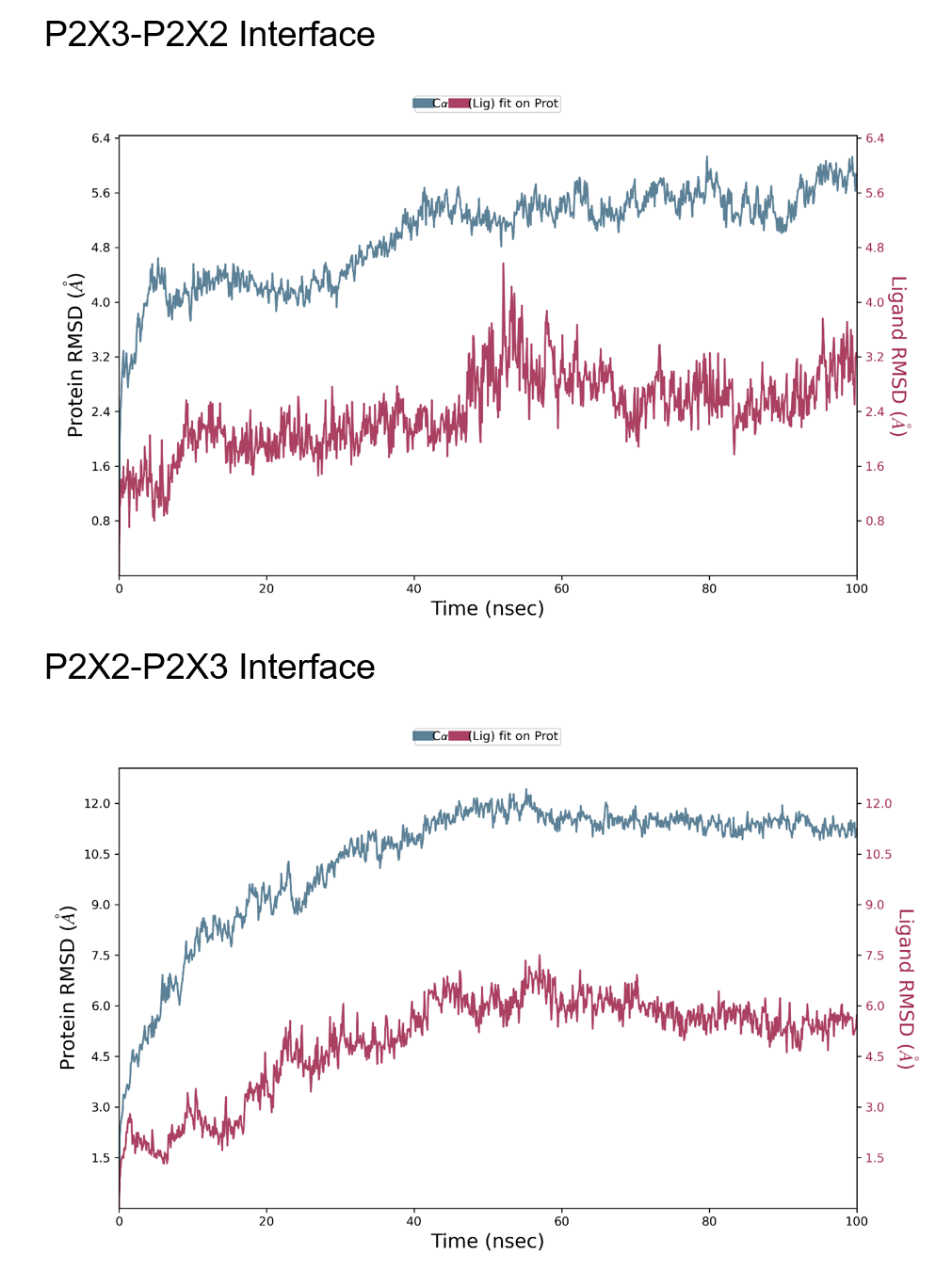


**Fig. S9. Molecular dynamic simulations of camlipixant-bound P2X2/3 receptor for Cam1 (top) and Cam2 (bottom).** The interactions are conducted for 100 ns. RMSD values are shown for both protein and each camlipixant ligand.

**
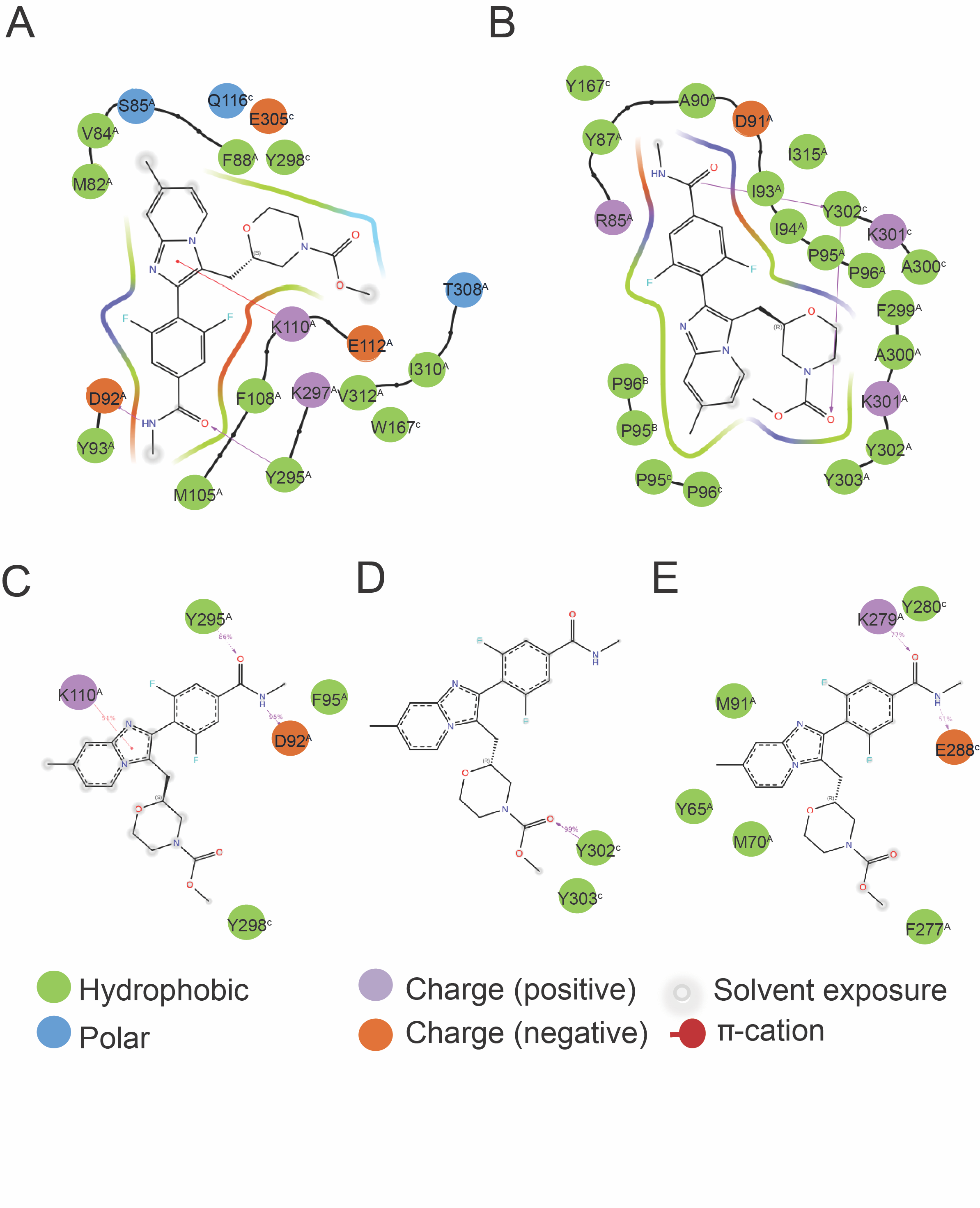
**

**Fig. S10. The Cam binding site appears across P2X receptors.** Cam occupies the same binding site with a similar pose and orientation in P2X3 (Fig. 3E), as observed in (A) P2X7 (PDB-ID, 5U1Y) and (B) P2X4 (PDB-ID, 8JV5). Molecular dynamic simulations of camlipixant-bound P2X receptor interactions are conducted for P2X7 (C), P2X4 (D), and P2X3 (E).


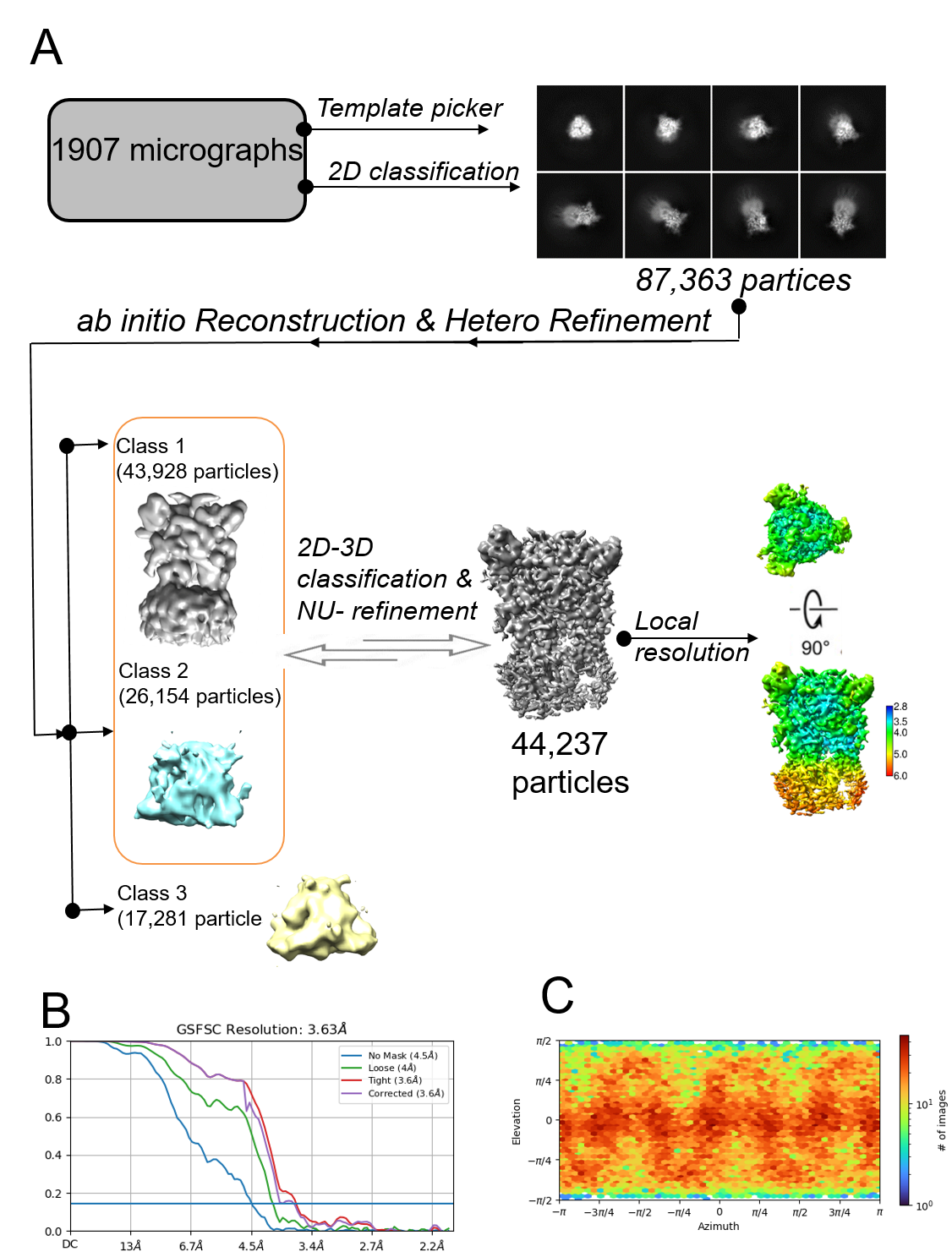


**Fig. S11. The cryo-EM data processing pipeline for P2X3:ATP complex**. (A) An overview of the processing steps, leading to a cryo-EM map with an overall resolution of 3.63 Å made with 50.6% of the initially exported particles. A local resolution map was calculated with cryoSPARC. This map indicates that the lowest resolution (depicted in red) correlates with the transmembrane helices, as observed in both side and top views from the extracellular side. The maps were visualized using UCSF ChimeraX. (B) Gold-standard Fourier shell correlation (FSC) curves were generated for resolution estimation. (C) The angular distribution of the particles used for the final map is illustrated.


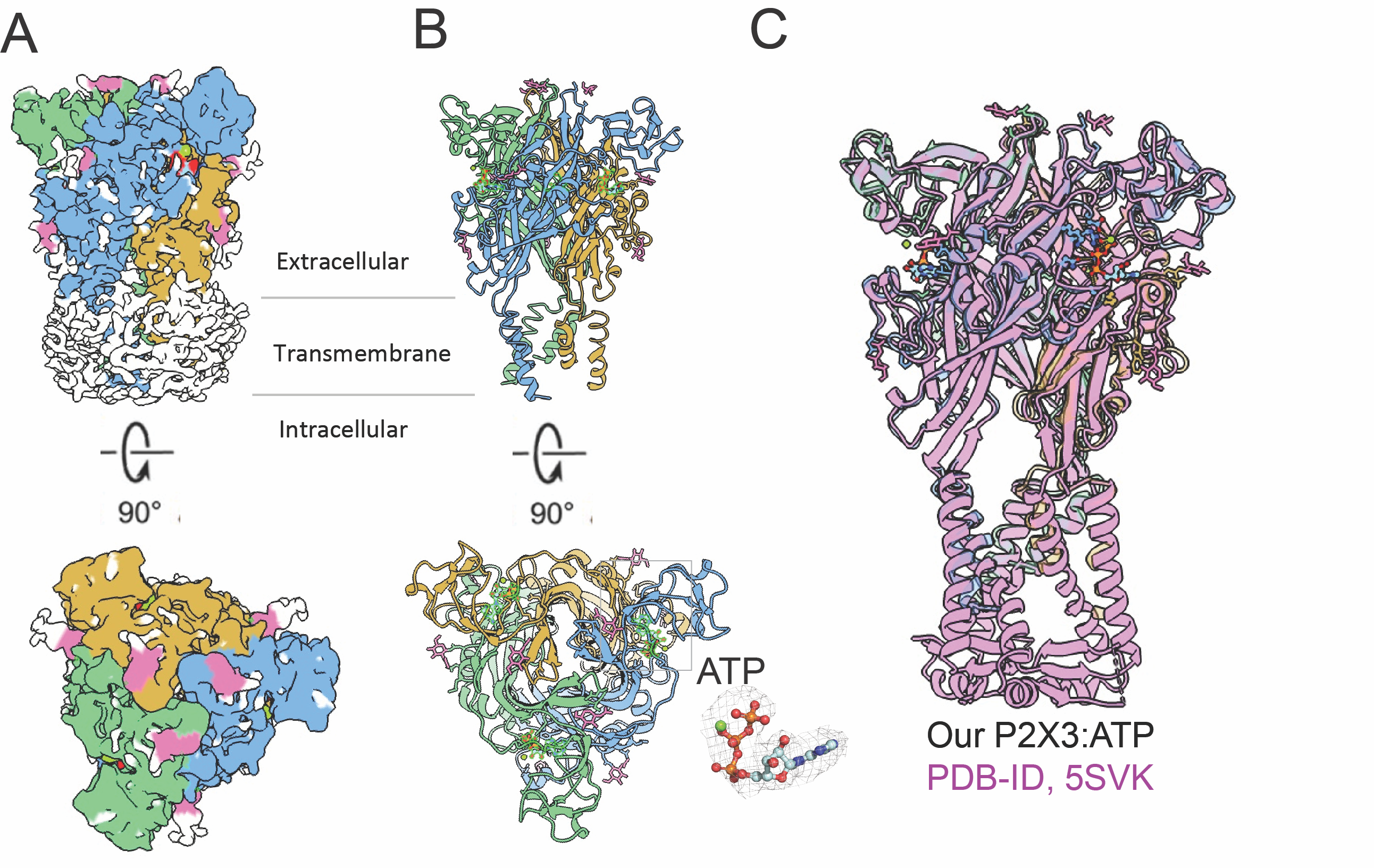


**Fig. S12. The cryo-EM structure of the P2X3:ATP complex.** Each subunit color-coded is presented with the protomers of the trimer-colored light blue, light orange, and green. (A) Representations of the cryo-EM map viewed parallel to the membrane (top panel) and perpendicular to the membrane from the extracellular side (bottom panel). (B) The structure is depicted as cartoons. ATP bound to the receptor is represented as stick-spheres, viewed parallel to the membrane (top panel) and perpendicular to the membrane from the extracellular side (bottom panel). The electron density map for ATP contoured at 3.0 σ. (C) The cryo-EM structure of ATP-bound P2X3 was superimposed with the crystal structure of P2X3:ATP. (highlighted in pink, PDB-ID, 5VSK). Cartoon representations illustrate both structures, revealing a high similarity with minimal conformational changes observed in the ATP-binding site within the upper body domain.


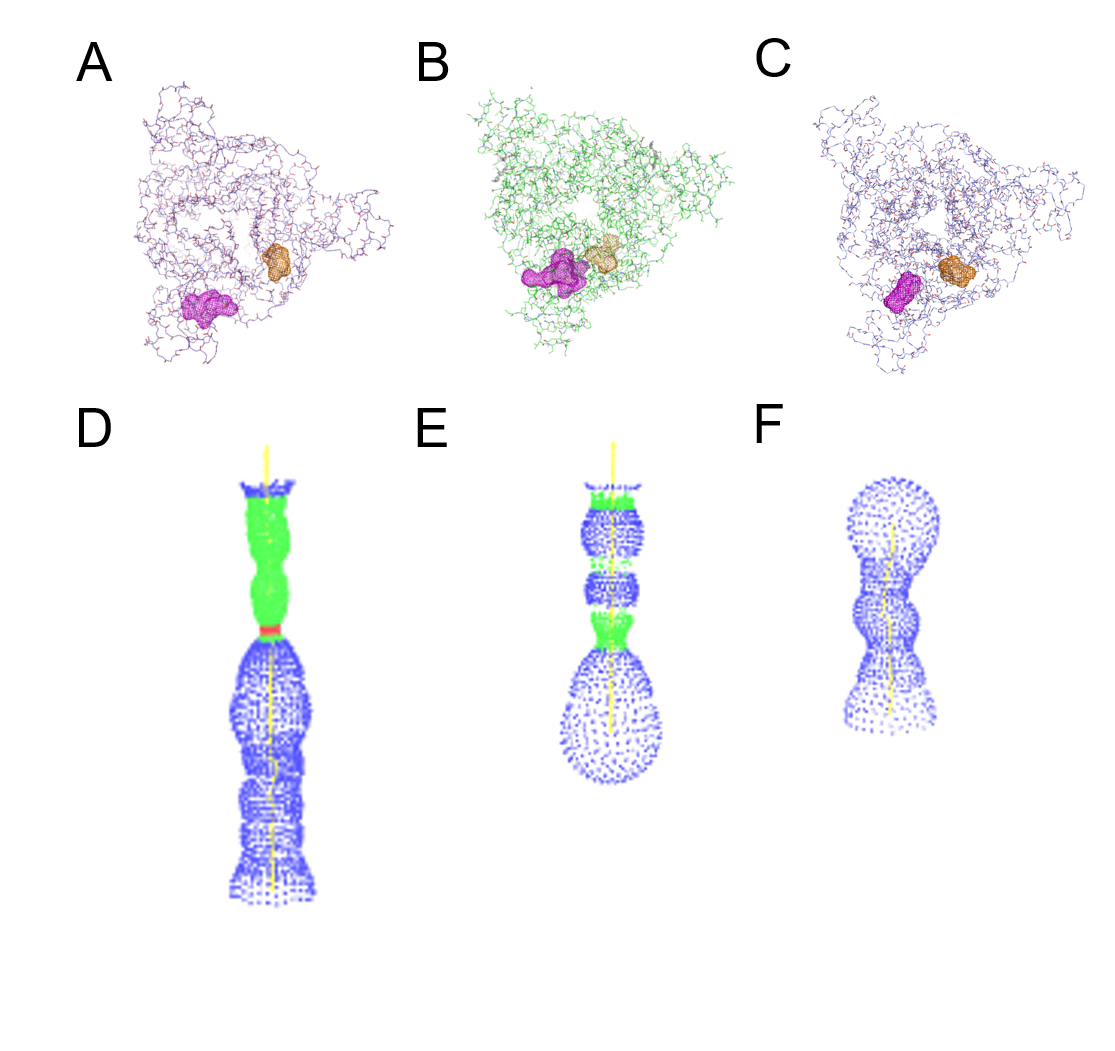


**Fig. S13. The drug-binding pocket enlarges in the camlipixant-bound P2X3 receptor.** (A) Line representation of the P2X3 structure, highlighting the ATP binding pocket (purple) and the Cam binding pocket (orange). The top views of P2X3:ATP (A), apo P2X3 (B), and P2X3:camlipixant (C) are displayed. (D-F) Dot representations of P2X3:ATP (D), apo P2X3 (E), and P2X3:camlipixant (F) illustrate the internal-space turret along the molecular threefold axis running through the center of the structures. These dot plots are generated using HOLE, where blue represents a radius greater than 2.3 Å, green indicates a radius between 1.15 and 2.3 Å, and red signifies a radius less than 1.0 Å.


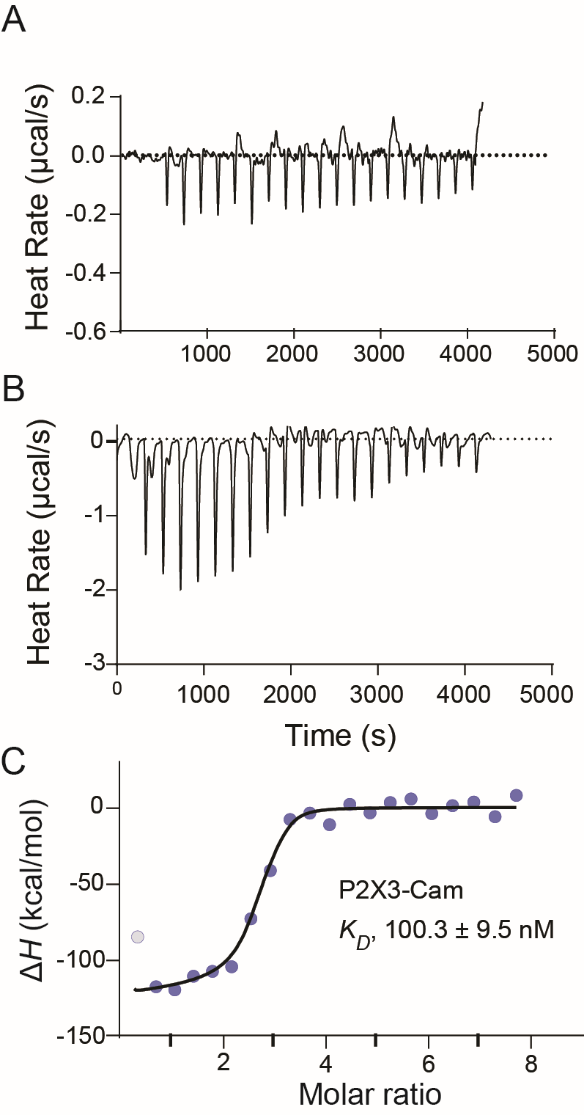


**Fig. S14. The binding affinity of camlipixant to P2X3 was determined using ITC**. Representative, ITC curves for titrations of Cam into either P2X3:ATP (A) or P2X3 apo (B), and integrated binding isotherms are shown (C).


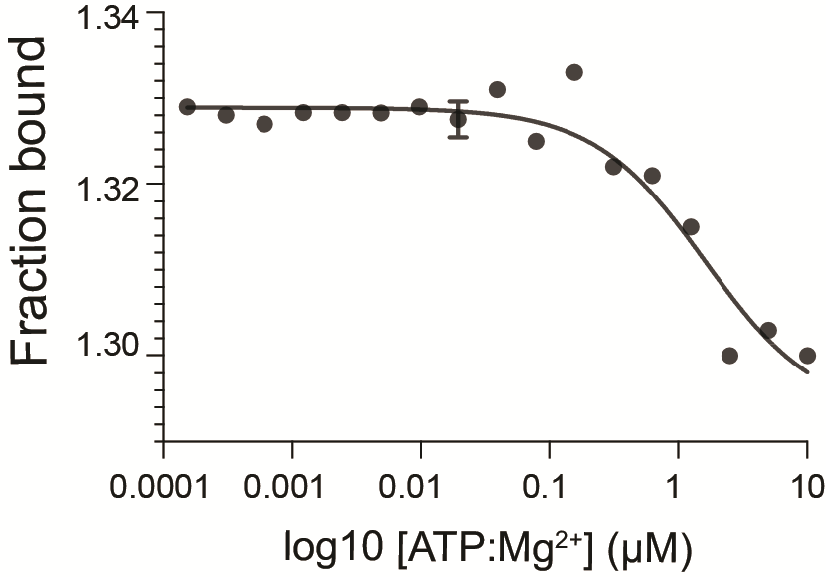


**Fig. S15. The binding affinity of ATP to P2X3 was determined using MST.** Integrated binding thermophoresis is displayed for the interaction. A representative binding thermophoresis displayed to show the binding nature of ATP toward P2X3.
